# The Efficacy of Electromagnetic Diathermy for the Treatment of Musculoskeletal Disorders: A Systematic Review with Meta-Analysis

**DOI:** 10.3390/jcm12123956

**Published:** 2023-06-09

**Authors:** Joel Pollet, Giorgia Ranica, Paolo Pedersini, Stefano G. Lazzarini, Simone Pancera, Riccardo Buraschi

**Affiliations:** IRCCS Fondazione Don Carlo Gnocchi, 20148 Milan, Italy; jpollet@dongnocchi.it (J.P.); ppedersini@dongnocchi.it (P.P.); slazzarini@dongnocchi.it (S.G.L.); spancera@dongnocchi.it (S.P.); rburaschi@dongnocchi.it (R.B.)

**Keywords:** musculoskeletal diseases, physical therapy modalities, diathermy

## Abstract

OBJECTIVE: This study aims to establish the effect of electromagnetic diathermy therapies (e.g., shortwave, microwave, capacitive resistive electric transfer) on pain, function, and quality of life in treating musculoskeletal disorders. METHODS: We conducted a systematic review according to the PRISMA statement and Cochrane Handbook 6.3. The protocol has been registered in PROSPERO: CRD42021239466. The search was conducted in PubMed, PEDro, CENTRAL, EMBASE, and CINAHL. RESULTS: We retrieved 13,323 records; 68 studies were included. Many pathologies were treated with diathermy against placebo, as a standalone intervention or alongside other therapies. Most of the pooled studies did not show significant improvements in the primary outcomes. While the analysis of single studies shows several significant results in favour of diathermy, all comparisons considered had a GRADE quality of evidence between low and very low. CONCLUSIONS: The included studies show controversial results. Most of the pooled studies present very low quality of evidence and no significant results, while single studies have significant results with a slightly higher quality of evidence (low), highlighting a critical lack of evidence in the field. The results did not support the adoption of diathermy in a clinical context, preferring therapies supported by evidence.

## 1. Background

Musculoskeletal disorders (MSDs) affect 1.71 billion people globally, with impressive financial costs for healthcare systems [1,2]. According to the WHO, the core strategy to reduce the constant rise of people suffering from MSDs is represented by rehabilitation [2]. The “Rehabilitation 2030: a call for action” initiative of the WHO further calls for ever greater integration of rehabilitation within health systems at all levels, both for communities and for hospital services [3].

Rehabilitation of MSDs is delivered by multi-professional teams. Interventions vary according to disorders and impairments; evidence-based treatments are not always common and shared, even within the same countries, where therapists perform different treatments to manage the same condition. However, non-specific rehabilitation interventions are common and performed in different countries. Among them, diathermy is used in different modalities by physicians in low- and middle-income countries as well as in high- and very-high-income countries for the treatment of MSDs [4,5,6].

Diathermy is identified by the U.S. Food & Drug Administration as a therapeutic modality that produces deep heating under the skin, muscles, and joints for therapeutic purposes. FDA classifies it into three forms: shortwave diathermy (SWD) [7], microwave diathermy (MWD) [8], and sonic therapy or ultrasound (US) [9]. The latter category was not considered in this review as the literature provides many studies on its effectiveness [10,11,12,13]. Recently, another diathermy therapy, based on electromagnetic current, has been introduced alongside these categories. It is known as capacitive resistive electric transfer (CRET) and it can be considered as longwave diathermy (LWD) [14], as the wave frequency used is relatively lower than those of SWD and MWD. The physiological effects of diathermy exploit the principles of thermotherapy, specifically: an increase in blood perfusion which facilitates tissue healing, a local increase of oxygen and nutrients, improved muscle contraction capacity, and a possible positive change in pain sensation [8,15]. Interesting studies have hypothesized that the benefits of topical heat therapy could also be mediated at a central level. Functional brain imaging research has revealed central effects of non-noxious skin warming, with increased activation of the posterior insula and thalamus of the brain, thereby providing pain relief [16].

The field of use of these therapies is wide, but mainly centred on MSDs [8,17,18]. However, there are some exceptions in recent studies reporting possible effects of the treatment in COVID-19 [19], or in the management of post-stroke spasticity [20]. In many countries, the use of diathermy for therapeutic purposes is widespread, yet there are no systematic reviews to date that discuss the efficacy of this therapy in patients with MSDs.

This systematic review aims to assess the effect of electromagnetic diathermy, primarily on pain and function, and secondarily on quality of life (QoL), patient-rated overall improvement, and adverse events in adults with MSDs. 

## 2. Methods 

This systematic review of literature was conducted following the Cochrane Handbook for Systematic Reviews of Interventions (Version 6.3) and the PRISMA Checklist 2020 [21]. The protocol of this review was registered in PROSPERO: CDR42021239466.

### 2.1. Type of Studies

We included published randomized controlled trials (RCTs) in English, Italian, Spanish, and Dutch. 

### 2.2. Type of Participants

Adults suffering from MSDs, with no age limitation, were included. MSDs were identified according to the definitions proposed in the MESH term definition, in the Emtree description, and according to the WHO definition of musculoskeletal conditions. 

### 2.3. Type of Interventions

SWD, MWD, and CRET were considered, compared with any other intervention, sham and placebo included, or with no treatment.

SWD produces deep heat of subcutaneous tissues by the oscillation of high frequency (usually at 13.56 or 27.12 MHz) electromagnetic fields, with the interposition of two condenser probes [7].

MWD, through electromagnetic waves (915–2456 MHz), stimulates the molecules within the target tissue, transforming electrical energy into heat. MWD is effective on tissues containing water. This therapy is usually applied with a single radiator [8].

CRET works through electric fields at relatively low frequencies, from 448 kHz to 1000 kHz. It uses two electrodes, a neutral plate, and an electrode with two possible modalities, capacitive or resistive. Typically, the capacitive one utilizes a frequency of 600 kHz that generates an increase in the superficial temperature with consequent vasodilatation and catabolic liquid reabsorption. Resistive modality is characterized by a frequency of 450 kHz and the generation of deep heating, with subsequent oxygenation of the treated tissue [14].

### 2.4. Exclusion Criteria

Pilot and cross-over studies were excluded. Studies performing interventions based on ultrasound therapy and diathermy interventions in athermal modality were excluded. 

Experimental ultrasound-based interventions were not considered given the considerable amount of reviews already available in the literature [10,11,12,13].

### 2.5. Outcome Measures

Primary outcomes were pain relief and change in function. Secondary outcomes were QoL changes, patient-rated overall improvement, and adverse events. Where multiple outcome measures were present, we analysed data from a single outcome measure according to a predetermined hierarchy (Supplementary File S1). 

The assessment time points considered were post-treatment (PT), short-term follow-up (ST) (≤1 month), intermediate-term follow-up (IT) (≤3 months), and long-term follow-up (LT) (>3 months).

### 2.6. Search Strategy

An experienced author (SGL) designed the search strategy across PubMed, Physiotherapy Evidence Database (PEDro), Cochrane Central Register of Controlled Trials (CENTRAL), EMBASE, and Cumulative Index to Nursing and Allied Health Literature (CINAHL), Table 1. The search was launched on 27 December 2022.

### 2.7. Other Sources

The references of the included records were screened for other articles of interest. The protocol studies retrieved and published in clinicaltrials.gov and the International Clinical Trials Registry Platform were screened, and the authors were contacted to check if registered trials were concluded and consequently published; if they were published, we screened the retrieved record for inclusion. 

### 2.8. Selection of the Studies

Two reviewers [JP and RB] independently screened the records for title, abstract, and full text using the software Rayyan [22]. Disagreements were solved with the consensus of the two reviewers, and a third author [SGL] was consulted in case of persistent disagreement. 

### 2.9. Data Extraction

Two reviewers [RB and JP] extracted the data in a predefined excel sheet. Data were extracted regarding the study, methods, participants, interventions, outcomes, and notes.

### 2.10. Risk of Bias Assessment 

‘Risk of bias tool 1.0′ was used to assess RCTs using the criteria recommended by Cochrane [23]. Two reviewers [RB and JP] independently assessed the risk of bias. A third reviewer [PP] was consulted in case of disagreement.

### 2.11. Measures of Treatment Effect

Standardized mean differences (SMD) with 95% confidence intervals (95% CI) were calculated for continuous data. Mean difference (MD) was calculated for pooled studies with the same outcome measure and non-pooled studies. 

### 2.12. Certainty of Evidence 

‘GRADE handbook for grading quality of evidence and strength of recommendations’ [24] and GRADEpro GDT Software (McMaster University and Evidence Prime, 2022) were used for assessing the certainty of evidence for the main outcomes of this review (i.e., pain relief and improvement in function).

### 2.13. Dealing with Missing Data

Where data were not extractable or not fully reported, corresponding authors were contacted. To retrieve data, when they were presented graphically, or with missing means, we used the methods proposed by Cochrane Handbook [25,26]. In the case of graphic data, we used the software “https://automeris.io/WebPlotDigitizer/ (accessed on 28 February 2023)” to extract the values. In the case of data presented as median and interquartile range or minimum and maximal value, the mean and standard deviation was calculated according to the method proposed by Wan et al. [27].

### 2.14. Data Synthesis

Data were summarized by MSDs. For each disorder, data were presented for the outcomes considered in this systematic review (i.e., pain relief, change in function, QoL, patient-rated overall improvement, and adverse events). Where possible, the results of the studies were pooled according to the type of diathermy utilized in the intervention (e.g., SWD, MWD, CRET), considering similar comparisons to reduce a source of heterogeneity.

## 3. Results

**Figure 1:**
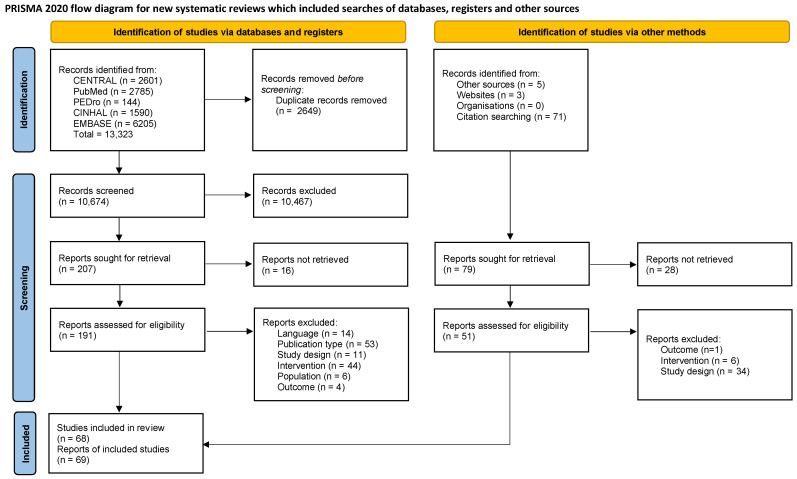
PRISMA flowchart [21].

The database search identified 13,323 records, and 79 extra records were identified through other methods. After the screening process, 69 reports of 68 studies were included. The full process has been synthesized in Figure 1. The 68 included studies considered 4892 patients affected by different MSDs. A certain degree of heterogeneity is evidenced in the studies regarding the types of proposed interventions. The diathermy with the highest occurrence was SWD, with 43 studies (63%) [28,29,30,31,32,33,34,35,36,37,38,39,40,41,42,43,44,45,46,47,48,49,50,51,52,53,54,55,56,57,58,59,60,61,62,63,64,65,66,67,68,69,70,71], and MWD had the second highest occurrence with 13 articles (19%) [72,73,74,75,76,77,78,79,80,81,82,83,84]. One article, Hammad 2019 [85], indifferently proposed SWD or MWD or hot packs under the label of thermotherapy as a treatment in addition to Kalternborn mobilization in patients with frozen shoulder.

We found 17 treated MSDs. The pathology most considered was OA, with 27 studies included in the review (40%), followed by LBP, with 12 studies (18%). 

**Figure 2:**
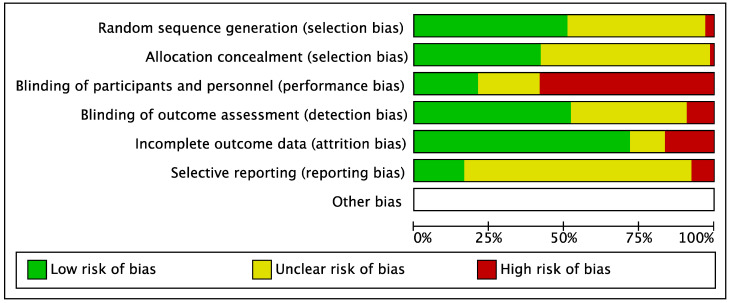
Risk of bias graph.

The risk of bias graphs (Figure 2 and Supplementary File S2) show for the selection bias that 46% of the studies did not report clearly how the random sequence was generated, and 56% did not report the allocation concealment. Furthermore, 57% of the studies had a high risk of bias in the blinding of participants and personnel, due to the difficulties in blinding in rehabilitation studies. Also, the assessor blinding had a low risk of bias in about half of the studies (51%). The outcome data were provided with a low risk of bias in 72% of the studies. The study protocol was coherent with the outcome measures presented in the paper in 12 studies (18%), whereas 7% of the studies modified the outcomes reported in the study protocol, and 75% of the studies did not present a study protocol. 

### 3.1. Knee and Hip Osteoarthritis

Twenty-seven studies [28,29,30,31,32,33,34,35,36,37,38,39,40,41,42,43,44,45,46,47,48,49,50,72,73,86,87,88] performed the treatment in adults with osteoarthritis (OA), 26 studies concerning knee OA, and 1 study concerning a mixed population affected by knee or hip OA. Of the 27 studies, 22 used SWD, 2 MWD, 2 CRET, and 1 ‘low power radiofrequency electromagnetic radiation’ (LPRER).

**Figure 3:**
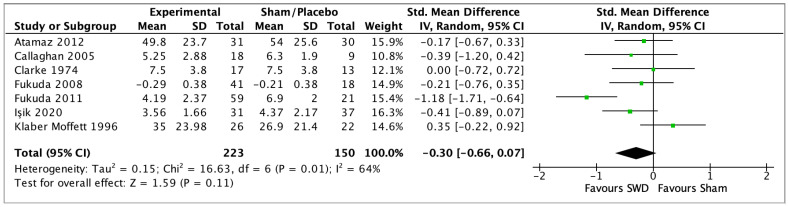
Forest plot of comparison: SWD vs. Sham (post treatment) in OA, outcome pain [29,30,31,32,33,35,36].

**Figure 4:**
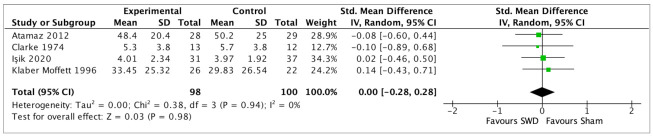
Forest plot of comparison: SWD vs. Sham (intermediate-term follow-up) in OA, outcome pain [29,30,35,36].

**Figure 5:**
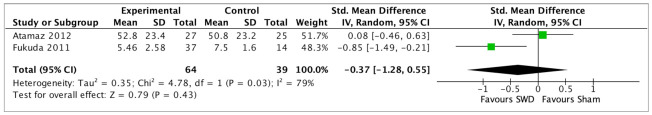
Forest plot of comparison: SWD vs. Sham (long term follow-up) in OA, outcome pain [30,32].

**Figure 6:**
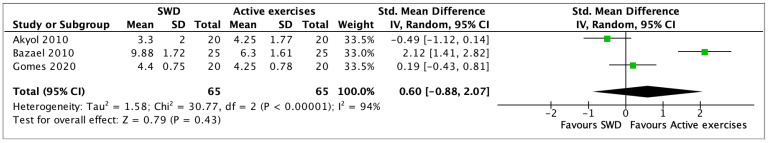
Forest plot of comparison: SWD vs. Active exercises (post treatment) in OA, outcome pain [37,39,40].

**Figure 7:**
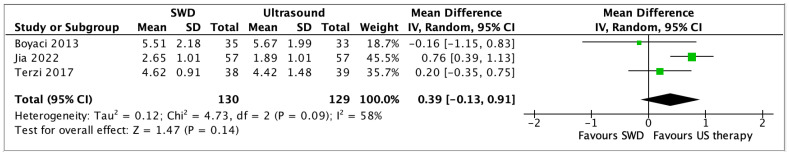
Forest plot of comparison: SWD vs. US therapy (post treatment) in OA, outcome pain [42,43,44].

**Figure 8:**
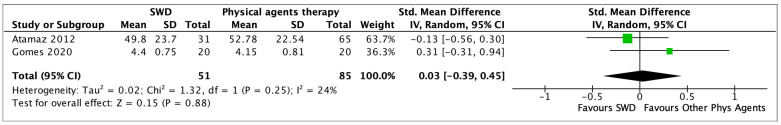
Forest plot of comparison: SWD vs. Other physical agent (post treatment) in OA, outcome pain [30,40].

**Figure 9:**
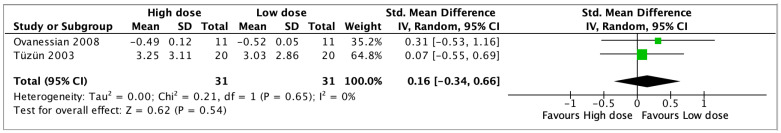
Forest plot of comparison: SWD high energy vs. SWD low energy (post treatment) in OA, outcome pain [47,49].

All 27 studies considered pain relief as an outcome. Eleven studies compared diathermy and a placebo or sham diathermy treatment (8 studies on SWD, 1 on MWD, and 2 on CRET). SWD was compared with sham treatment in 7 studies [29,30,31,32,33,34,35,36], the post-treatment assessments were pooled (SMD −0.30, 95% CI −0.66 to 0.07, random-effects model) with non-significant results (*p* = 0.11) and a heterogeneity, I^2^, of 64% (GRADE: low certainty), Figure 3. While Rattachaiyanot 2008 [28] did not present analysable data and reported no difference between sham SWD and SWD treatment for VAS pain scale, Wright 1964 [46] observed no differences in SWD treatment with respect to placebo treatments (based on tablets or injections). In the intermediate follow-up, 4 studies [29,30,34,35,36] were pooled (SMD 0.00, 95% CI −0.28 to 0.28, random-effects model) with a non-significant result (*p* = 0.98), with 0% of heterogeneity (GRADE: very low certainty), Figure 4. For the long-term follow-up, 2 studies [30,32] were pooled (SMD −0.37, 95% CI −1.28 to 0.55, random-effects model) with a non-significant result (*p* = 0.43), and a heterogeneity of 79% (GRADE: very low certainty), Figure 5. Four studies [37,38,39,40] compared SWD with a treatment based on active exercises; 3 studies [37,39,40] were pooled (SMD 0.60, 95% CI −0.88 to 2.07, random-effects model) with a non-significant result in the post-treatment (*p* = 0.43), and 94% of heterogeneity (GRADE: very low certainty), Figure 6. Chamberlain 1982 [38] showed no significant differences between the two interventions at each assessment, post-treatment, and intermediate follow-up for the VAS pain scale. The follow-up results of Akyol 2010 and Bezalel 2010 [37,39] are reported in Table 2. Four studies [41,42,43,44] compared SWD with US therapy; 3 studies [42,43,44] were pooled (MD 0.39, 95% CI −0.13 to 0.91, random-effects model) with non-significant results (*p* = 0.14) and a 58% heterogeneity for the post-treatment assessment (GRADE: very low), Figure 7. Cetin 2008 [41] showed no statistically significant differences after treatment between the two interventions for the VAS pain scale. The follow-up results of Terzi 2017 [42] and Jia 2022 [42,43] are reported in Table 2. Three studies [30,40,41] compared SWD with other physical agent therapies (see table of contents for the specific treatment of each of the included studies, Supplementary File S3. In the post-treatment assessment, 2 studies [30,40] were pooled (SMD 0.03, 95% CI −0.39 to 0.45, random-effects model) with non-significant results (*p* = 0.88), and a heterogeneity of 24% (GRADE: low certainty), Figure 8. Cetin 2008 [41] reported a non-significant difference between the two interventions for the VAS pain scale. The follow-up results of Atamaz 2012 [30] are shown in Table 2. Two studies [47,49] compared the treatment effects of different energy dosages (high energy dose compared with low energy dose) of SWD. The studies were pooled (SMD 0.16, 95% CI −0.34 to 0.66, random-effects model) with non-significant differences between the two groups (*p* = 0.54) and 0% of heterogeneity (GRADE: very low), Figure 9. Coccetta 2018 [86] compared CRET with a sham CRET treatment, but reported only graphically a significant reduction in pain intensity post-treatment, at short- and medium-term follow-ups for the VAS pain scale within groups. However, Cocetta 2018 did not report the results between groups. All the non-pooled comparison values of MD are presented in Table 2. 

**Figure 10:**
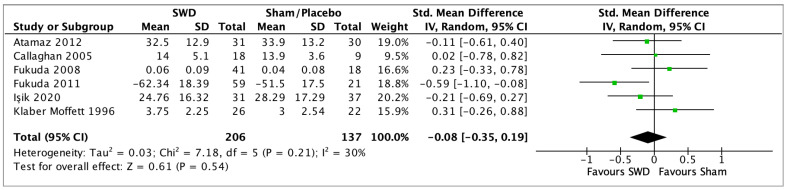
Forest plot of comparison: SWD vs. Sham (post treatment) in OA, outcome function [29,30,31,32,33,34].

**Figure 11:**
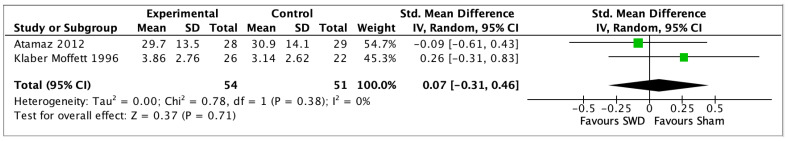
Forest plot of comparison: SWD vs. Sham (intermediate-term follow-up) in OA, outcome function [30,34].

**Figure 12:**
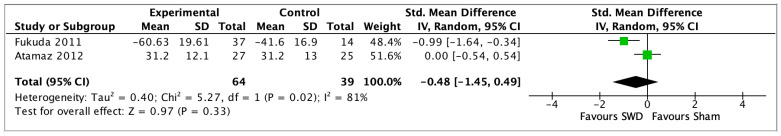
Forest plot of comparison: SWD vs. Sham (long term follow-up) in OA, outcome function [30,32].

**Figure 13:**
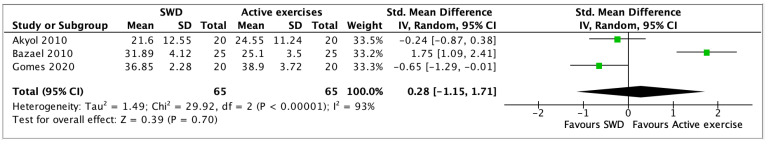
Forest plot of comparison: SWD vs. Active exercises (post treatment) in OA, outcome function [37,39,40].

**Figure 14:**
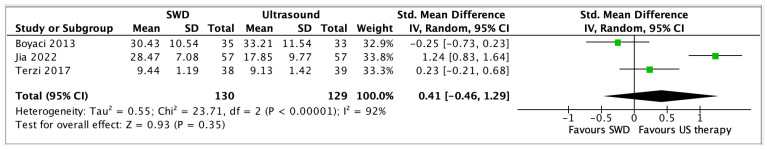
Forest plot of comparison: SWD vs. US therapy (post treatment) in OA, outcome function [42,43,44].

**Figure 15:**
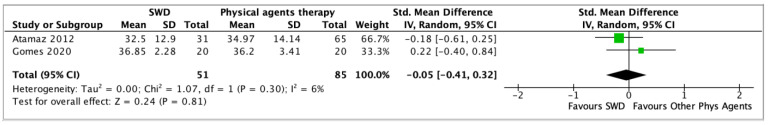
Forest plot of comparison: SWD vs. Other physical agent (post treatment) in OA, outcome function [30,40].

**Figure 16:**
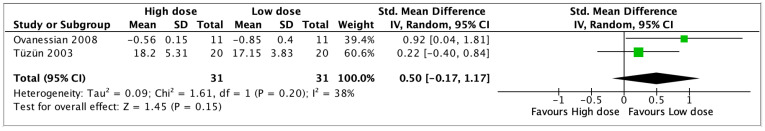
Forest plot of comparison: SWD high energy vs. SWD low energy (post treatment) in OA, outcome function [47,49].

Twenty-six studies [28,29,30,31,32,33,34,35,36,37,38,39,40,41,42,43,44,45,47,48,49,50,72,73,86,87,88] assessed function as an outcome of their interventions. SWD was compared with placebo/sham SWD in 8 studies. Six of the studies [29,30,31,32,33,34,35] were pooled (SMD −0.08, 95% CI −0.35 to 0.19, random-effects model) with a non-significant result (*p* = 0.54), and I^2^ of 30% for the PT assessment (GRADE: low), Figure 10. Clarke 1974 [36] reported only pooled data between SWD and sham SWD patients, so it was not considered in the analysis at each time point. At IT follow up, 2 studies [30,34,35] were pooled (SMD 0.07, 95% CI −0.31 to 0.46, random-effects model) with a non-significant result (*p* = 0.40), and 0% of heterogeneity for (GRADE: very low), Figure 11. At LT follow-up, 2 studies [30,32] were pooled (SMD −0.48, 95% CI −1.45 to 0.49, random-effects model), with non-significant results (*p* = 0.33), and I^2^ = 81% (GRADE: very low), Figure 12. Three studies [37,39,40] compared the effect of SWD to active exercises in PT (SMD 0.28, 95% CI −1.15 to 1.71, random-effects model), with non-significant results (*p* = 0.70) and I^2^ = 93% (GRADE: very low), Figure 13. The follow-up values are shown in Table 3. Four studies [41,42,43,44] compared the effect of SWD with those of US therapy; 3 studies [42,43,44] were pooled (SMD 0.41, 95% CI −0.46 to 1.29, random-effects model) with a non-significant result (*p* = 0.35), and I^2^ = 92% post-treatment (GRADE: very low), Figure 14. Cetin 2008 [41] reported no differences between the two therapies for the Lequense Index. Follow-up values are shown in Table 3. Three studies [30,40,41] evaluated the functional improvements comparing SWD and other physical agent therapies; 2 [30,40] of them were pooled (SMD −0.05, 95% CI −0.41 to 0.32, random-effects model) with non-significant results (*p* = 0.81) and I^2^ = 6% (GRADE: Low), Figure 15. Cetin 2008 reported a significant improvement of function between pre- and PT within groups, but no differences were found between SWD and the other physical agent therapy considered for the Lequense index. The follow-up results of Atamaz 2012 [30] are reported in Table 3. Two studies [47,49] compared different energy doses of SWD in the treatment of knee OA. The data were pooled (SMD 0.50, 95% CI −0.17 to 1.17, random-effects model) with non-significant results (*p* = 0.15), and heterogeneity of I^2^ = 38% (GRADE: very low), Figure 16. Clarke 1974 [36] provided only aggregate data and no *p*-value for the differences between SWD and sham SWD and for the comparison between SWD and ice application, so it was not possible to evaluate the effectiveness of the intervention. All the non-pooled comparison values of MD are presented in Table 3.

Six studies [29,32,39,45,49,50] assessed the QoL level in patients with knee OA who underwent diathermy treatments. The pooled data of 2 studies [29,32] comparing SWD and sham SWD (SMD 0.55, 95% CI 0.20 to 0.90, random-effects model) showed a significant result (*p* = 0.002) in favour of SWD therapy, and no heterogeneity (I^2^ = 0%). All the non-pooled comparison values of MD are presented in Table 4. Ovanessian 2008 [49] compared high- and low-energy SWD, and reported no difference between groups for the KOOS (Knee Injury and Osteoarthritis Outcome Score) QoL subscale. 

Three studies [42,44,45] assessed the patient-reported overall improvement; 2 studies [42,44] comparing SWD and US therapy were pooled (SMD 0.03, 95% CI −0.30 to 0.36, random-effects model) with no significant differences between the interventions (*p* = 0.86), and no heterogeneity (I^2^ = 0%). Cantarini 2006 [45] reported no differences between SWD and routine PT evaluated by an overall efficacy assessment (a scale from 0 to 4 points).

The GRADE assessment for the certainty of evidence for the main outcomes considered in the studies in patients with OA ranges from low to very low.

### 3.2. Low Back Pain

Twelve studies [51,52,53,54,55,56,57,74,75,89,90,91] proposed treatment for Low Back Pain (LBP) utilizing four different diathermy therapies; SWD in 7 studies, MWD in 2 studies, and CRET in 3 studies.

**Figure 17:**
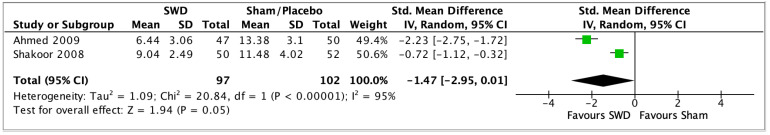
Forest plot of comparison: SWD vs. Sham (post treatment) in LBP, outcome pain [52,55].

**Figure 18:**
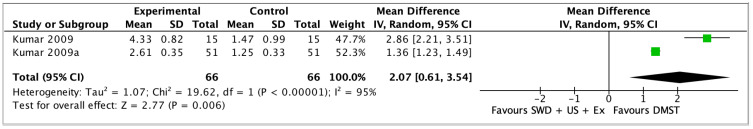
Forest plot of comparison: SWD + US therapy + Lumbar strengthening exercises vs. Dynamic Muscular Stabilization Techniques (post treatment) in LBP, outcome pain [53,54].

Two studies [52,55] compared SWD with sham SWD. They were pooled (SMD −1.47, 95% CI −2.95, 0.01, random-effects model) with non-significant results (*p* = 0.05) and I^2^ of 95% (GRADE: very low), Figure 17. Two studies [53,54] compared conventional therapy (designed as SWD, US therapy, and lumbar strengthening exercises) with Dynamic Muscular Stabilization Techniques (DMST) were pooled (MD 2.07, 95% CI 0.61, 3.54, random effects model), with results in favour of DMST for VAS (*p* = 0.006), and I^2^ of 95% (GRADE: very low), Figure 18. Non-pooled data for pain relief of Durmus 2014 [74] did not show significant changes in favour of MWD + active exercises vs. active exercise only at any time point. Non-pooled data for pain relief of Igatpurkiar 2013 and Ansari 2022 [51,57] showed significant changes in favour of the control group, respectively: Maitland mobilization + hot packs + core stabilization at post-treatment (MD 0.60, 95% CI 0.23 to 0.97, random-effects model) and Graeco-Arabic massage at post-treatment (MD 2.50, 95% CI 1.50 to 3.50, random-effects model). In three studies [89,90,91], non-pooled data for pain relief showed significant important changes in favour of CRET. Specifically, non-pooled data for pain relief in Zati 2018’s study [89] highlighted significant changes in favour of CRET deep heating (MD −0.90, 95% CI −1.57 to −0.23, random-effects model) vs. superficial heating post-treatment. Non-pooled data for pain relief in Notarnicola 2017 [90] found significant changes in favour of CRET vs. Laser at Short-Term follow-up (MD −1.90, 95% CI −2.85 to −0.95, random-effects model), while Wachi 2022 [91] found significant changes in favour of CRET compared with sham CRET at post-treatment (MD −3.30, 95% CI −4.12 to −2.48, random-effects model) (Table 5). Gibson 1985 [56] assessed the effectiveness of SWD, placebo SWD (i.e., detuned SWD), and osteopathy. All the treatments reported an improvement within groups (*p* < 0.01) for VAS daytime and nocturnal pain score, both after treatment and at IT. A comparison between groups was not presented. Farrell 1982 [75] compared passive mobilization and manipulation with MWD plus isometric abdominal exercises and ergonomic instructions. The results for pain (mean subjective rating, from 0 to 10 points) were reported graphically and showed a trend toward pain reduction in both groups, with no significant difference between the two groups. 

Eight studies [51,53,54,56,57,74,89,90] assessed improvement in function in patients with Low LBP. Non-pooled data for improvement in function of Kumar 2009/2009a [53,54] revealed significant changes in favour of the dynamic muscular stabilization technique group compared with SWD + ultrasound + lumbar strengthening exercises post-treatment. Moreover, non-pooled data for Ansari 2022 [57] showed significant improvement for the control Graeco-Arabic massage group (MD 3.80, 95% CI 0.73 to 6.87, random-effects model) compared with SWD post-treatment. Non-pooled data of three studies [51,56,90] revealed significant improvement in function, in favour respectively of: SWD post-treatment (MD 0.80, 95% CI 0.09 to 1.51, random-effects model), SWD + traction + core stabilization post-treatment (MD −5.70, 95% CI −10.94 to −0.46, random-effects model), and CRET at short-term follow-up (MD −17.40, 95% CI −26.20 to −8.60, random-effects model). Non-pooled data for improvement in function in Durums 2014, Zati 2018 [74,89] and the comparison of SWD vs. Osteopathy in the Gibson 1985 study [56] showed no significant changes in favour of any treatment groups at any time point (Table 6). Farrell 1982 [75] compared passive mobilization and manipulation with MWD plus isometric abdominal exercises and ergonomic instructions. An improvement in lumbar extension was reported for the manipulation and mobilization group (*p* < 0.05), while no other significative improvement in lumbar motion was reported. Wachi 2022 [91] compared CRET with sham CRET, calculating the differences in muscle time onset during manual muscle tests. The results showed a significant decrease in onset time in three out of four muscles in the CRET group.

Only the non-pooled data of the Durmus 2014 study compared the effects of diathermy + active exercises vs. only active exercises on the QoL, and did not find significant changes in favour of any of the two groups (Table 7).

The GRADE assessment for the certainty of evidence for the main outcomes considered in the studies in patients with LBP ranges from low to very low.

### 3.3. Shoulder Tendinopathies (STN) 

Six studies [58,59,76,77,78,92] evaluated the efficacy of diathermy for treating STN. Two studies utilized SWD, 3 studies used MWD, and 1 utilized CRET. All 6 studies assessed pain relief. Non-pooled data for pain relief in Yilmaz Kaysin’s 2018 study [58] showed significant changes in favour of SWD compared with sham SWD at the short-term follow-up (MD −1.64, 95% CI −2.98 to 0.31, random-effects model) and at the intermediate follow-up (MD −2.10, 95% CI −3.48 to 0.73, random-effects model). Similarly, non-pooled data for pain in Giombini’s 2006 study [78] underlined significant changes in favour to MWD compared with active exercises at post-treatment (MD −2.90, 95% CI −3.35 to −2.45, random-effects model) and at intermediate-term follow-up (MD −3.70, 95% CI −4.32 to −3.08, random-effects model). In the same study, a comparison between MWD vs. ultrasound therapy showed significant changes in pain relief in non-pooled data, in favour of the MWD post-treatment (MD −3.40, 95% CI −3.99 to −2.81, random-effects model) and at intermediate-term follow-up (MD −2.95, 95% CI −3.54 to −2.36, random-effects model). In contrast, non-pooled data for pain relief in Rabini’s 2012 study [77] reported significant changes in favour of the control subacromial corticosteroid injections group, compared with MWD at long-term follow-up (MD 9.50, 95% CI 1.70 to 17.30, random-effects model). Non-pooled data for pain relief in Jimenez-Garcia 2008 [59], Akyol 2012 [76], and Avendaño-Coy 2022 [92] did not show any significant changes in favour of any considered groups at any time point (Table 8).

All 6 studies assessed improvements in function. Non-pooled data for improvement in function in Yilmaz Kaysin’s 2018 study revealed significant changes in favour of SWD compared with sham SWD at the short-term follow-up (MD 10.48, 95% CI –0.56 to 15.52, random-effects model) and at the intermediate follow-up (MD 14.15, 95% CI 6.26 to 22.04, random-effects model). Similarly, non-pooled data for improvement in function in Giombini’s 2006 study found significant changes in favour to MWD comparing it with active exercises at post-treatment (MD 16.90, 95% CI 13.54 to 20.26, random-effects model) and at intermediate-term follow-up (MD 18.73, 95% CI 14.28 to 23.18, random-effects model). In the same study, a comparison between MWD vs. ultrasound therapy showed significant changes in improvement in function in favour of MWD post-treatment (MD 18.10, 95% CI 15.24 to 20.96, random-effects model) and at intermediate-term follow-up (MD 20.25, 95% CI 16.43 to 24.07, random-effects model). In contrast, non-pooled data for improvement in function in Akyol’s 2012 study [76] reported significant changes in favour of the control sham MWD group compared with MWD at post-treatment (MD −2.35, 95% CI −3.50 to −1.20, random-effects model) and short-term follow-up (MD −4.05, 95% CI −5.23 to −2.87, random-effects model). Non-pooled data for improvement in function in Jimenez-Garcia 2008, Rabini 2012, and Avendaño-Coy 2022 [59,77,92] did not show any significant changes in favour of any considered groups at any time point (Table 9).

Akyol 2012 and Avendaño-Coy 2022 assessed QoL improvement, but did not underline any significant changes in favour of any considered groups at any time point (Table 10).

The GRADE assessment for the certainty of evidence for the main outcomes considered in the studies in patients with STN ranges from low to very low.

### 3.4. Frozen Shoulder (FS)

Three studies [60,61,85] evaluated the effect of diathermy in the treatment of the frozen shoulder. Two studies [60,61] compared SWD with other interventions, while Hammad 2019 [85] evaluated the effect of adding diathermy treatment (MWD or SWD) to a manual therapy intervention (i.e., Kalternborn mobilization). Only Guler-Uysal 2008 [60] assessed patients’ pain relief post-treatment and non-pooled data highlighted significant changes in favour of the control Cyriax treatment + other interventions (MD 12.10, 95% CI 0.03 to 24.17, random-effects model) compared with SWD + hot packs + other interventions (Table 11). In the same study, the authors assessed improvement in function and non-pooled data showed significant changes, also in this case, in favour of the control group (MD −21.60, 95% CI −33.93 to −9.27, random-effects model). In contrast, non-pooled data for improvement in function post-treatment in Hammad’s 2019 study showed significant changes in favour of diathermy + Kaltenborn mobilization (MD −51.80, 95% CI −54.94 to 48.66, random-effects model) compared with only Kaltenborn mobilization. In addition, non-pooled data for improvement in function in Leung’s 2008 study [61] showed no significant changes post-treatment and at short-term follow up comparing SWD + stretching exercises vs. hot packs (+ stretching exercises). In contrast, the same study presented significant changes in favour of the SWD + stretching exercises group, comparing it with only stretching exercise post-treatment (MD 21.70, 95% CI 9.47 to 33.93, random-effects model) and at short-term follow-up (MD 17.50, 95% CI 1.76 to 33.24, random-effects model) (Table 12).

The GRADE assessment for the certainty of evidence for the main outcomes considered in the studies in patients with FS is very low.

### 3.5. Carpal Tunnel Syndrome (CTS)

**Figure 19:**
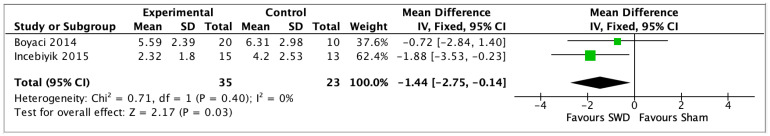
Forest plot of comparison: SWD vs. Sham (post treatment) in CTS, outcome pain [62,63].

**Figure 20:**
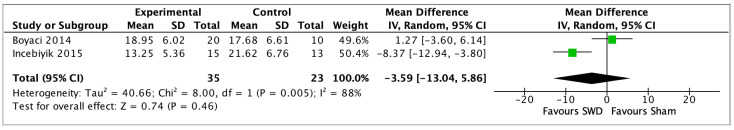
Forest plot of comparison: SWD vs. Sham (post treatment) in CTS, outcome function [62,63].

Three studies [62,63,79] proposed interventions based on diathermy to treat CTS; two of them used SWD, the other MWD. All studies assessed pain relief. The studies of Boyaci 2014 and Incebiyik 2015 [62,63] compared the effects of SWD and sham SWD on the VAS scale. Their results were pooled (MD −1.44, 95% CI −2.75 to −0.14, random-effects model) with a significant reduction in pain (*p* = 0.03) in favour of SWD, with I^2^ = 0. (GRADE: low), Figure 19. Frasca 2011 [79] compared MWD with sham MWD, reporting a significant reduction in pain for the MWD intervention group within and between groups for the VAS pain scale. All of the three studies retrieved assessed functional improvements. The data of Boyaci 2014 and Incebiyik 2015, regarding the Boston Carpal Tunnel Questionnaire (Functional status), were pooled (MD −3.59, 95% CI −13.04 to 5.86, random-effects model), with no differences (*p* = 0.46), and I^2^ = 88%. (GRADE: very low), Figure 20. Frasca 2011 compared MWD with Sham MWD and found no difference both within groups and between groups for the Levine Boston Questionnaire part II. 

The GRADE assessment for the certainty of evidence for the main outcomes considered in the studies in patients with CTS ranges from low to very low.

### 3.6. Lower Limb Tendinopathies (LLT)

Two studies [80,81] treated LLT with diathermy (MWD). Giombini 2002 [80] included athletes with Achilles and patellar tendinopathies, while Cheng 2018 [81] included athletes with patellar tendinopathies. In this contest, non-pooled data from Giombini 2002 showed significant changes post-treatment in pain relief in the MWD group (MD −2.20, 95% CI −3.09 to −1.11, random-effects model) compared with ultrasound therapy. In contrast, Cheng 2018 showed significant changes in favour of the control extracorporeal shock wave therapy (MD 3.70, 95% CI 3.12 to 4.28, random-effects model) compared with MWD + acupuncture + ultrasound therapy (Table 13). Non-pooled data for improvement in function in the Cheng 2018 study did not find significant important changes in any of the considered groups (Table 14).

The GRADE assessment for the certainty of evidence for the main outcomes considered in the studies in patients with LLT is very low.

### 3.7. Neck Pain (NP)

Two studies [64,82] evaluated the effect of diathermy in the treatment of NP: Dziedzic 2005 [64] with SWD, and Ortega 2013 [82] with MWD. Neither of the two studies showed significant differences in favour of any groups considered, at any time point, and in any outcomes assessed: pain relief, improvement in function, and quality of life (Table 15, Table 16 and Table 17). Dziedzic 2005, and Ortega 2013 reported no differences in the patient-reported overall improvement for the proposed interventions.

The GRADE assessment for the certainty of evidence for the main outcomes considered in the studies in patients with NP ranges from low to very low.

### 3.8. Patellofemoral Pain (PFP)

Two studies [65,93] verified the effect of diathermy on treating PFP. Albornoz-Cabello 2020 [93] used monopolar dielectric radiofrequency, and Verma 2012 [65] used SWD. 

Verma 2012 reported significant relief in both groups (SWD + active exercises vs. taping + active exercises) but did not compare the results of the two interventions. Moreover, this study showed a significant improvement in function in both groups without comparing the two interventions. Non-pooled data of the Albornoz-Cabello 2020 study highlighted significant changes post-treatment in favour of monopolar dielectric radiofrequency + active exercise in pain relief (MD −53.00, 95% CI −59.22 to −46.78, random-effects model), and improvement in function (MD 22.00, 95% CI 15.45 to 28.55, random-effects model) compared with only active exercise (Table 18 and Table 19).

The GRADE assessment for the certainty of evidence for the main outcomes considered in the studies in patients with PFP is very low.

### 3.9. Temporomandibular Joint (TMJ)

Two studies [66,67] treated TMJ problems with SWD and compared it with other treatments. Specifically, Talaat 1986 [67] did not show significant changes in pain relief comparing SWD vs. ultrasound therapy, while they showed significant changes post-treatment in favour of SWD by comparing it with treatment with a tablet of methocarbamol + acetyl salicylic acid (MD −1.12, 95% CI −1.49 to −0.75, random-effects model) (Table 20). Gray 1995 [66] compared different treatments, namely SWD, Megapulse, US therapy, laser therapy, and a placebo treatment. The reported results were a mix of patient-reported improvement and non-specified objective measurements. Data were reported in absolute and relative frequencies. No significant differences were retrieved among the four interventions, but all four treatments showed a significant improvement compared to the placebo treatment.

The GRADE assessment for the certainty of evidence for the main outcomes considered in the studies in patients with TMJ is very low.

### 3.10. Delayed Onset of Muscular Soreness (DOMS)

Two studies [94,95] utilized diathermy to treat DOMS. Visconti 2020 [94] assessed the effect of CRET for the treatment of DOMS in athletes, while Nakamura 2022 [95] treated healthy subjects with DOMS with CRET comparing it with no treatment. Notably, non-pooled data in Visconti’s 2020 study showed no significant effect in either group on pain relief (Table 21). Futhermore, they reported no differences in the global impression of change (*p* = 0.638) among the CRET, Sham CRET, and Massage groups. Nakamura 2022 showed no significant changes comparing CRET vs. no intervention in improvement in function (Table 22).

The GRADE assessment for the certainty of evidence for the main outcomes considered in the studies in patients with DOMS is low.

### 3.11. Humerus Fractures

The study of Livesley 1992 [68] compared the effect of SWD combined with a standard physiotherapy treatment (specific contents were not described), with sham SWD combined with the same standard physiotherapy treatment. This study showed no differences in pain relief and improvement in function between the two interventions.

### 3.12. Ulnar Nerve Entrapment (UNE)

Badur 2020 [69] compared SWD with sham SWD in patients with UNE. No significant results in favour of any of the groups were found in the considered outcomes: pain relief, improvement in function, and QoL (Table 23, Table 24 and Table 25).

The GRADE assessment for the certainty of evidence for the main outcomes considered in this study in patients with UNE is low.

### 3.13. Lateral Epicondylitis (LE)

Babaei-Ghazani 2019 [70] compared SWD and sham SWD with the addition of transverse friction massage, stretching, strengthening, and education intervention in the treatment of patients with LE. Non-pooled pain relief data showed significant effects in favour of SWD post-treatment (MD −26.30, 95% CI −32.60 to −20.00, random-effects model) and at intermediate-term follow-up (MD −21.20, 95% CI −26.11 to −16.29, random-effects model) (Table 26). Additionally, non-pooled data for improvement in function showed significant effects in favour of SWD post-treatment (MD −21.20, 95% CI −28.52 to −13.88, random-effects model) and at intermediate-term follow-up (MD −17.20, 95% CI −23.39 to −11.01, random-effects model) (Table 27).

The GRADE assessment for the certainty of evidence for the main outcomes considered in this study in patients with LE is low.

### 3.14. Ankle or Foot Sprain

The study of Pasila 1978 [71] compared two different devices administering pulsed SWD therapy with sham SWD treatment. No significant differences were reported among the three interventions (adduction and abduction strength of the forefoot, ankle range of motion) except for the gait impairment score, for which one pulsed SWD machine (Diapulse) was significantly more effective in solving gait impairment.

### 3.15. Lower Limb Acute Muscle Injury (LAMI)

Giombini 2001 [83] compared the effect of MWD and US therapy in subjects affected by LAMI at different muscles of the lower limbs (i.e., biceps femoris, adductors, quadriceps, and gastrocnemius). Non-pooled data of pain relief in LAMI (Table 28) reveals significant effects in favour of MWD post-treatment (MD −2.20, 95% CI −2.90 to −1.50, random-effects model). 

The GRADE assessment for the certainty of evidence for the main outcomes considered in this study in patients with LAMI is very low.

### 3.16. Tension-Type Headache (TTH)

Georgoudis 2017 [84] investigated the effect of myofascial release, MWD, stretching, and acupuncture versus stretching and acupuncture in patients with TTH. The authors reported no time*treatment interaction on VAS average. A pre-post improvement for pain relief (VAS average) was graphically reported for both groups. 

### 3.17. Total Knee Replacement (TKR)

García-Marín 2021 [96] studied TKR post-operative pain. All three groups underwent usual physiotherapy (active mobilization, strengthening, and walking), and then one group underwent CRET while the other performed sham CRET. No significant results in favour of any of the three groups were found in the considered outcomes: pain relief, improvement in function, and QoL (Table 29, Table 30 and Table 31).

The GRADE assessment for the certainty of evidence for the main outcomes considered in this study in patients with TKR is low.

## 4. Discussion

This systematic review aimed to evaluate the effectiveness of electromagnetic diathermy for treating MSDs to reduce pain and improve function. The role of diathermy within treatment protocols was found to be very varied. It was proposed as a stand-alone therapy, especially when compared with sham intervention, as a component of multimodal treatment, or even considered within the usual care intervention. Consequently, diathermy was proposed within the experimental and control groups. 

Diathermy was used as a treatment in 17 different MSDs. Both acute and chronic conditions were treated, based on the positive effect that thermotherapy can add to the treatment of these conditions [97,98]. However, in seven conditions only a single study was performed to prove the effectiveness of therapy. In only five MSDs, three or more studies were included. This limits the possibility to provide final conclusions on the topic.

In those MSDs where only few studies could be pooled, high levels of heterogeneity were retrieved, even if the manageable sources of heterogeneity were considered. This can represent a sign of deficiency in the study conduction of some of the primary studies.

Other authors have performed systemic reviews on diathermy in MSD treatment. Contrary to our results, Wang et al. [17] reported the efficacy of SWD against sham or no intervention in patients with knee OA for pain relief. It is worth pointing out that, in the meta-analysis by Wang et al., studies that did not have a placebo or no treatment as a control intervention were aggregated (Cetin 2008 and Cantarini 2006 [41,45]). In our meta-analysis, on the other hand, only the comparison of SWD versus placebo or sham was considered. We also included our major source of heterogeneity (Fukuda 2011 [32]), removing which would have changed the I^2^ from 64% to 0%, but would not have changed the pooled result. In addition, Wang et al. combined the placebo and no-treatment groups, as in Fukuda 2011, whereas we did not consider them two different interventions. 

Other reviews [18,99] report a possible efficacy of CRET for pain relief and improvement in function in a mixed population, also including patients with MSDs. Their results should be interpreted considering the different study designs included (e.g., cases series and non-RCT studies), as well as the wide choice of outcome indicators and the lack of an assessment of the certainty of the evidence.

This study is the first systematic review that has assessed the effect of different types of electromagnetic diathermies on MSDs. Even if the pathologies, outcome, and the different types of diathermies considered create a huge number of results, the adopted methodology, and the methods of conducting were used to provide a confident response. 

It is well known that therapies based on heat, including electromagnetic diathermies, are widely adopted all around the world [4,5,6], but the underlying evidence supporting their adoption is not so strong. Clinicians should focus on therapies supported by stronger evidence and use diathermies when—through their evaluation—benefits could be produced by heat. 

Different studies included in this review provide clear, reliable, and encouraging results supporting diathermy treatments. However, the results of these studies should be confirmed by other trials, with large sample sizes and appropriate study designs.

This review has some limitations; it did not provide a sensitivity analysis of the results. This is because the wide number of studies and pathologies included did not allow for such analysis. Further studies should investigate the specific pathologies and perform this analysis. Another limit of this review is that it did not show a strong clinical implication, even if in the treatment of knee OA meta-analysis results showed clearly that SWD is not effective. In some of the MSDs where more studies were retrieved, the unclear use of diathermy treatments with disparate treatment did not allow an extensive pooling of study results. Moreover, in other MSDs this review highlights the lack of evidence, with only single studies that provide limited results. 

## 5. Conclusions

In conclusion, the findings of our review are influenced by the scarce quality of evidence. Further studies should perform trials with a larger sample size, experimental interventions based on diathermy as a stand-alone therapy to reduce the complexity of multi-approach protocols, control interventions defined according to MSDs guidelines, and a reduction of sequence generation and allocation bias. 

The studies published up to now, even if providing a low quality of evidence, do not allow us to suggest the use of diathermy in clinical settings or its wide implementation within rehabilitative protocols. Indeed, there is no strong evidence that diathermy is preferable to placebo/sham intervention or other interventions for treating MSDs, even if in some specific cases diathermy showed significant results. 

## Figures and Tables

**Table 1:** Search strategy.

Database	Search Strategy
MEDLINE (Pubmed)	Diathermy [Mesh] OR radiowaves [Mesh] OR hyperthermia [Mesh] “Tecar”[Title/Abstract] OR “radiofrequency treatment”[Title/Abstract] OR “capacitive resistive”[Title/Abstract] OR “capacitive and resistive”[Title/Abstract] OR “electric transfer”[Title/Abstract] OR “deep heating”[Title/Abstract] OR “CRET” [Title/Abstract] OR “SWD”[Title/Abstract] OR “shortwave diathermy”[Title/Abstract] OR “short-wave diathermy”[Title/Abstract] OR “MWD”[Title/Abstract] OR “microwave diathermy”[Title/Abstract] OR “micro-wave diathermy”[Title/Abstract] #1 OR #2 Randomized controlled trial”[pt] OR “controlled clinical trial”[pt] OR “randomized”[tiab] OR “placebo”[tiab] OR “clinical trials as topic”[mesh:noexp] OR “randomly”[tiab] OR “trial”[ti] Animals [mh] NOT humans [mh] #4 NOT #5 #3 AND #6
PEDro	Tecar, method: clinical trial Radiofrequency, method: clinical trial Capacitive AND resistive, method: clinical trial Electric AND transfer, method: clinical trial Deep AND heating, method: clinical trial Diathermy, method: clinical trial Radiowaves, method: clinical trial Hyperthermia, method: clinical trial
Cochrane Central Register of Controlled Trials	MeSH descriptor: [diathermy] explode all trees MeSH descriptor: [radio waves] explode all trees MeSH descriptor: [hyperthermia] explode all trees (“Tecar” OR “radiofrequency treatment” OR “(capacitive NEAR/6 resistive)” OR “electric transfer” OR “deep heating” OR “diathermy” OR “radiowaves” OR “hyperthermia”):ti,ab,kw
EMBASE	Tecar:ti,ab,kw OR (‘radiofrequency treatment’/exp NOT ‘radiofrequency ablation’/exp) OR ((radiofrequency NEAR/3 (treatment* OR therap*)):ti,ab,kw) OR ((capacitive NEAR/3 resistive):ti,ab,kw) OR ‘electric transfer’:ti,ab,kw OR ‘deep heating’:ti,ab,kw OR ‘diathermy’/exp OR ‘diathermy’:ti,ab,kw OR ‘radiowaves’:ti,ab,kw OR ‘thermotherapy’/exp OR ‘thermotherapy’:ti,ab,kw (‘Randomized controlled trial’/de OR ‘controlled clinical trial’/de OR random*:ti,ab OR ‘randomization’/de OR ‘intermethod comparison’/de OR placebo:ti,ab OR compare:ti OR compared:ti OR comparison:ti OR ((evaluated:ab OR evaluate:ab OR evaluating:ab OR assessed:ab OR assess:ab) AND (compare:ab OR compared:ab OR comparing:ab OR comparison:ab)) OR ((open NEXT/1 label):ti,ab) OR (((double OR single OR doubly OR singly) NEXT/1 (blind OR blinded OR blindly)):ti,ab) OR ‘double blind procedure’/de OR ((parallel NEXT/1 group*):ti,ab) OR crossover:ti,ab OR ‘cross over’:ti,ab OR (((assign* OR match OR matched OR allocation) NEAR/6 (alternate OR group OR groups OR intervention OR interventions OR patient OR patients OR subject OR subjects OR participant OR participants)):ti,ab) OR assigned:ti,ab OR allocated:ti,ab OR ((controlled NEAR/8 (study OR design OR trial)):ti,ab) OR volunteer:ti,ab OR volunteers:ti,ab OR ‘human experiment’/de OR trial:ti) NOT (((random* NEXT/1 sampl* NEAR/8 (‘cross section*’ OR questionnaire* OR survey OR surveys OR database OR databases)):ti,ab) NOT (‘comparative study’/de OR ‘controlled study’/de OR ‘randomised controlled’:ti,ab OR ‘randomized controlled’:ti,ab OR ‘randomly assigned’:ti,ab) OR (‘cross-sectional study’ NOT (‘randomized controlled trial’/de OR ‘controlled clinical study’/de OR ‘controlled study’/de OR ‘randomised controlled’:ti,ab OR ‘randomized controlled’:ti,ab OR ‘control group’:ti,ab OR ‘control groups’:ti,ab)) OR (‘case control*’:ti,ab AND random*:ti,ab NOT (‘randomised controlled’:ti,ab OR ‘randomized controlled’:ti,ab)) OR (‘systematic review’:ti NOT (trial:ti OR study:ti)) OR (nonrandom*:ti,ab NOT random*:ti,ab) OR ‘random field*’:ti,ab OR ((‘random cluster’ NEAR/4 sampl*):ti,ab) OR (review:ab AND review:it NOT trial:ti) OR (‘we searched’:ab AND (review:ti OR review:it)) OR ‘update review’:ab OR ((databases NEAR/5 searched):ab) OR ((rat:ti OR rats:ti OR mouse:ti OR mice:ti OR swine:ti OR porcine:ti OR murine:ti OR sheep:ti OR lambs:ti OR pigs:ti OR piglets:ti OR rabbit:ti OR rabbits:ti OR cat:ti OR cats:ti OR dog:ti OR dogs:ti OR cattle:ti OR bovine:ti OR monkey:ti OR monkeys:ti OR trout:ti OR marmoset*:ti) AND ‘animal experiment’/de) OR (‘animal experiment’/de NOT (‘human experiment’/de OR ‘human’/de))) #1 AND #2
CINAHL	(MH “Diathermy+”) OR (MM “radio waves”) OR (MH “hyperthermia, induced+”) OR TI (“tecar” OR “radiofrequency treatment” OR “capacitive resistive” OR “capacitive and resistive” OR “electric transfer” OR “deep heating” OR “diathermy” OR “radiowaves” OR “hyperthermia”) OR AB(“Tecar” OR “radiofrequency treatment” OR “(capacitive N6 resistive)” OR “electric transfer” OR “deep heating” OR “diathermy” OR “radiowaves” OR “hyperthermia”) OR SU(“Tecar” OR “radiofrequency treatment” OR “(capacitive N6 resistive)” OR “electric transfer” OR “deep heating” OR “diathermy” OR “radiowaves” OR “hyperthermia”) AND ((MH “randomized controlled trials”) OR (MH “double-blind studies”) OR (MH “single-blind studies”) OR (MH “random assignment”) OR (MH “pretest-posttest design”) OR (MH “cluster sample”) OR TI(randomised OR randomized) OR AB(random*) OR TI(trial) OR ((MH “sample size”) AND AB(assigned OR allocated OR control)) OR (MH “placebos”) OR PT(“randomized controlled trial”) OR AB(control W5 group) OR (MH “crossover design”) OR (MH “comparative studies”) OR AB(cluster W3 RCT)) NOT (((MH “animals+”) OR (MH “animal studies”) OR TI(animal model*)) NOT (MH “human”))

**Table 2:** Non-pooled data for OA pain relief.

Author Year	Assessment Time	Outcome Measure	MD Value	95% CI	Significantly in Favour of	GRADE
**SWD vs. Active exercises**						
Bezalel 2010 [37]	ST	WOMAC pain subscale	4.76	3.82 to 5.70	Active exercises	⨁⨁◯◯ Low
Akyol 2010 [39]	IT	VAS	0.30	−1.66 to 2.26	//	⨁◯◯◯ Very low
**SWD vs. Ultrasound therapy**						
Terzi 2017 [42]	ST	VAS	−0.47	−0.90 to −0.04	SWD	⨁◯◯◯ Very low
Jia 2022 [43]	IT	VAS	−0.11	−0.47 to 0.25	//	⨁⨁◯◯ Low
	LT	VAS	1.30	0.93 to 1.63	Ultrasound therapy	⨁⨁◯◯ Low
**SWD vs. Other physical agent therapy**						
Atamaz 2012 [30]	IT	VAS	−0.58	−10.26 to 9.10	//	⨁◯◯◯ Very low
	LT	VAS	5.12	−5.71 to 15.95	//	
**SWD vs. Photobiomodulation**						
Gomes 2020 [40]	PT	NPRS	0.20	−0.35 to 0.75	//	⨁◯◯◯ Very low
SWD vs. Ice						
Clarke 1974 [36]	PT	Likert scale	2.70	0.06 to 5.34	Ice	⨁◯◯◯ Very low
**SWD vs. Phonophoresis**						
Boyaci 2013 [44]	PT	VAS	0.48	−0.43 to 1.39	//	⨁◯◯◯ Very low
**SWD vs. Routine ambulatory care**						
Cantarini 2006 [45]	PT	VAS	−25.14	−39.19 to −11.09	SWD	⨁◯◯◯ Very low
	IT	VAS	−22.04	−40.24 to −3.84	SWD	
**SWD + Other physical agents therapy vs. Intra-articular injections**						
Atamaz 2006 [50]	PT	VAS	−9.95	−18.10 to −1.80	SWD	⨁◯◯◯ Very low
	IT	VAS	−5.05	−11.13 to 1.03	//	
	LT	VAS	6.65	−2.16 to 15.46	//	
**SWD continuous mode vs. SWD pulsed mode**						
Teslim 2013 [48]	PT	NPRS	−0.91	−1.68 to −0.14	SWD continuous mode	⨁⨁◯◯ Low
**MWD vs. Sham MWD**						
Giombini 2010 [72]	PT	WOMAC pain subscale	−7.40	−9.35 to −5.45	MWD	⨁⨁◯◯ Low
	IT	WOMAC pain subscale	−8.00	−10.28 to −5.72	MWD	
**LPRER vs. TENS**						
Alcidi 2007 [88]	PT	VAS	−3.00	−19.79 to 13.79	//	⨁◯◯◯ Very low
	ST	VAS	1.25	−15.17 to 17.66	//	
**CRET vs. Sham CRET**						
Kumaran 2019 [87]	PT	VAS	−1.50	−2.32 to −0.67	CRET	⨁◯◯◯ Very low
	ST	VAS	−1.68	−3.13 to −0.23	CRET	
	IT	VAS	−1.04	−2.90 to 0.82	//	

CRET: Capacitive Resistive Electric Transfer; IT: Intermediate-Term follow-up; LPRER: Low Power Radiofrequency Electromagnetic Radiation; LT: Long-Term follow-up; MWD: Microwave Diathermy; NPRS: Numeric Pain Rating Scale; PT: Post Treatment; ST: Short-Term follow up; SWD: Shortwave Diathermy; TENS: Trans-cutaneous Electrical Nerve Stimulation; VAS: Visual Analogue Scale; WOMAC: Western Ontario and McMaster Universities Osteoarthritis Index.

**Table 3:** Non-pooled data for OA improvement in function.

Author Year	Assessment Time	Outcome Measure	MD Value	95% CI	Significantly in Favour of	GRADE
**SWD vs. Sham SWD**						
Rattanachaiyanont 2008 [28]	PT	WOMAC physical function subscale	−0.11	−0.57 to 0.80	//	⨁⨁◯◯ Low
**SWD vs. active exercises**						
Bezalel 2010 [37]	ST	WOMAC physical function subscale	12.35	10.06, 14.46	Active exercises	⨁⨁◯◯ Low
Akyol 2010 [39]	IT	WOMAC physical function subscale	−0.20	−10.17 to 9.77	MWD	⨁◯◯◯ Very low
**SWD vs. Ultrasound therapy**						
Terzi 2017 [42]	ST	Lequesne Index	0.24	−0.24 to 0.72	//	⨁◯◯◯ Very low
Jia 2022 [43]	IT	WOMAC total score	7.57	4.54 to 10.60	Ultrasound therapy	⨁⨁◯◯ Low
	LT	WOMAC total score	6.96	3.85 to 10.07	Ultrasound therapy	⨁⨁◯◯ Low
**SWD vs. Other physical agent therapy**						
Atamaz 2012 [30]	IT	WOMAC physical function subscale	−3.85	−10.01 to 2.31	//	⨁◯◯◯ Very low
	LT	WOMAC physical function subscale	−1.76	−7.66 to 4.14	//	
**SWD vs. Photobiomodulation**						
Gomes 2020 [40]	PT	WOMAC physical function subscale	−2.35	−3.71 to −0.99	SWD	⨁◯◯◯ Very low
**SWD vs. Phonophoresis**						
Boyaci 2013 [44]	PT	WOMAC physical function subscale	−0.81	−5.17 to 3.55	//	⨁◯◯◯ Very low
**SWD vs. Routine ambulatory care**						
Cantarini 2006 [45]	PT	Lequesne Index	−3.34	−6.07 to −0.61	SWD	⨁◯◯◯ Very low
	IT	Lequesne Index	−1.47	−4.08 to 1.14	//	
**SWD + Other physical agents therapy vs. Intra-articular injections**						
Atamaz 2006 [50]	PT	WOMAC physical function subscale	−0.05	−5.86 to 5.761.80	//	⨁◯◯◯ Very low
	IT	WOMAC physical function subscale	−0.05	−5.90 to 5.80	//	
	LT	WOMAC physical function subscale	−0.05	−5.56 to 5.46	//	
**SWD continuous mode vs. SWD pulsed mode**						
Teslim 2013 [48]	PT	Active knee flexion ROM	12.65	5.88 to 19.42	SWD continuous mode	⨁⨁◯◯ Low
**MWD vs. Sham MWD**						
Giombini 2010 [72]	PT	WOMAC physical function subscale	−30.90	−37.77 to −24.03	MWD	⨁⨁◯◯ Low
	IT	WOMAC physical function subscale	−33.30	−40.77 to −25.83	MWD	
**CRET vs. Sham CRET**						
Kumaran 2019 [87]	PT	WOMAC total score	−0.77	−1.51 to −0.02	CRET	⨁◯◯◯ Very low
	ST	WOMAC total score	−12.33	−22.92 to −1.74	CRET	
	IT	WOMAC total score	−4.27	−17.58 to 9.04	//	

CRET: Capacitive Resistive Electric Transfer; IT: Intermediate-Term follow-up; LT: Long-Term follow-up; MWD: Microwave Diathermy; PT: Post Treatment; ST: Short-Term follow-up; SWD: Shortwave Diathermy; WOMAC: Western Ontario and McMaster Universities Osteoarthritis Index.

**Table 4:** Non-pooled data for QoL outcome in OA.

Author Year	Assessment Time	Outcome Measure	MD Value	95% CI	Significantly in Favour of
**SWD vs. Sham SWD**					
Işik 2020 [29]	IT	SF-36—General Health subscale	2.75	−4.26 to 9.76	//
Fukuda 2011 [32]	LT	Knee injury and osteoarthritis outcome score-QoL subscale	3.37	−5.24 to 11.98	//
**SWD vs. Active exercises**					
Akyol 2010 [39]	PT	SF-36—General Health subscale	4.25	−4.49 to 12.99	//
	IT	SF-36—General Health subscale	0.50	−9.36 to 10.36	//
**SWD vs. Routine ambulatory care**					
Cantarini 2006 [45]	PT	Arthritis impact measurement scale	−0.16	−0.45 to 0.13	//
	IT	Arthritis impact measurement scale	−0.33	−0.65 to −0.01	SWD
**SWD + Other physical agents therapy vs. Intra-articular injections**					
Atamaz 2006 [50]	PT	SF-36—Physical functioning subscale	10.50	0.33 to 20.67	SWD
	IT	SF-36—Physical functioning subscale	−2.00	−11.82 to 7.82	//
	LT	SF-36—Physical functioning subscale	1.90	−7.12 to 10.92	//

IT: Intermediate-Term follow-up LT: Long-Term follow-up PT: Post Treatment; SWD: Shortwave Diathermy.

**Table 5:** Non-pooled data for pain relief in LBP.

Author Year	Assessment Time	Outcome Measure	MD Value	95% CI	Significantly in Favour of	Grade
**MWD + active exercises vs. active exercises**						
Durmus 2014 [74]	PT	VAS	−0.34	−1.32, 0.64	//	⨁◯◯◯ Very low
	ST	VAS	−0.21	−0.21, 0.68	//	
**SWD + traction + core stabilization vs. Maitland mobilization + hot packs + core stabilization**						
Igatpurkiar 2013 [51]	PT	VAS	0.60	0.23, 0.97	Maitland mobilization + hot packs + core stabilization	⨁◯◯◯ Very low
**SWD vs. Graeco-Arabic massage**						
Ansari 2022 [57]	PT	VAS	2.50	1.50, 3.50	Graeco-Arabic massage	⨁⨁◯◯ Low
**CRET deep heating vs. CRET superficial heating**						
Zati 2018 [89]	PT	NPRS	−0.90	−1.57, −0.23	CRET deep heating	⨁◯◯◯ Very low
	ST	NPRS	−0.70	−1.85, 0.45	//	
**CRET vs. Laser**						
Notarnicola 2017 [90]	PT	VAS	0.10	−0.97, 1.17	//	⨁◯◯◯ Very low
	ST	VAS	−1.90	−2.85, −0.95	CRET	
**CRET vs. Sham CRET**						
Wachi 2022 [91]	PT	VAS	−3.30	−4.12, −2.48	CRET	⨁⨁◯◯ Low

CRET: Capacitive Resistive Electric Transfer; MWD: Microwave Diathermy; NPRS: Numeric Pain Rating Scale; PT: Post Treatment; ST: Short-Term follow up; SWD: Shortwave Diathermy; VAS: Visual Analogue Scale.

**Table 6:** Non-pooled data for improvement in function in LBP.

Author Year	Assessment Time	Outcome Measure	MD Value	95% CI	Significantly in Favour of	Grade
**SWD + Ultrasound + Lumbar strengthening exercises vs. Dynamic Muscular Stabilization Techniques**						
Kumar 2009 [53]	PT	Stair climbing [number/min]	5.74	3.07, 8.41	Dynamic Muscular Stabilization Techniques	⨁◯◯◯ Very low
Kumar 2009 [54]	PT	BPC [mmHg]	11.35	10.15, 12.55	Dynamic Muscular Stabilization Techniques	
	PT	APC [mmHg]	6.57	5.96, 7.18	Dynamic Muscular Stabilization Techniques	
**SWD vs. Sham SWD**						
Gibson 1985 [56]	PT	Lumbar spine flexion ^+^	0.80	0.09, 1.51	SWD	⨁◯◯◯ Very low
	IT	Lumbar spine flexion ^+^	0.60	−0.26, 1.46	//	
**SWD vs. Osteopathy**						
Gibson 1985 [56]	PT	Lumbar spine flexion ^+^	0.20	−0.46, 0.86	//	⨁◯◯◯ Very low
	IT	Lumbar spine flexion ^+^	0.30	−0.50, 1.10	//	
**SWD + traction + core stabilization vs. Maitland mobilization + hot packs + core stabilization**						
Igatpurkiar 2013 [51]	PT	ODI	−5.70	−10.94, −0.46	SWD + traction + core stabilization	⨁◯◯◯ Very low
**SWD vs. Graeco-Arabic massage**						
Ansari 2022 [57]	PT	ODI	3.80	0.73, 6.87	Graeco-Arabic massage	⨁⨁◯◯ Low
**MWD + active exercises vs. Active exercises**						
Durmus 2014 [74]	PT	ODI	−0.47 *	−3.22, 2.28	//	⨁◯◯◯ Very low
	ST	ODI	−1.52 *	−4.35, 1.31	//	
**CRET (deep heating vs. superficial heating)**						
Zati 2018 [89]	PT	ODI	−0.50	−8.18, 7.18	//	⨁◯◯◯ Very low
	ST	ODI	−3.80	−11.05, 3.45	//	
**CRET vs. Laser therapy**						
Notarnicola 2017 [90]	PT	ODI	−6.40	−13.95, 1.15	//	⨁◯◯◯ Very low
	ST	ODI	−17.40	−26.20, −8.60	CRET	

APC: Abdominal Pressure Change; BPC: Back Pressure Change; CRET: Capacitive Resistive Electric Transfer; IT: Intermediate-Term follow up; LPRER: Low Power Radiofrequency Electromagnetic Radiation; MWD: Microwave Diathermy; ODI: Oswestry Disability Index; PT: Post Treatment; ST: Short-Term follow up; SWD: Shortwave Diathermy; ^+^ Macrae and Wright method; * Value expressed as Delta (Post Treatment—Before Treatment; Follow-up—Before Treatment).

**Table 7:** Non-pooled data for quality of life in LBP.

Author Year	Assessment Time	Outcome Measure	MD value	95% CI	Significantly in Favour of
**MWD + active exercises vs. Active exercises**					
Durmus 2014 [74]	PT	SF-36 general health subscale *	0.82	−7.62, 9.26	//
	ST	SF-36 general health subscale *	0.68	−6.57, 7.93	//

MWD: Microwave Diathermy; PT: Post Treatment; ST: Short-Term follow up; * Value expressed as Delta (Post Treatment—Before Treatment; Follow-up—Before Treatment); SF-36: Short Form Health Survey 36.

**Table 8:** Non-pooled data for pain relief in STN.

Author Year	Assessment Time	Outcome Measure	MD Value	95% CI	Significantly in Favour of	GRADE
**SWD** (+ conservative treatment program) vs. **Sham SWD **(+ conservative treatment program)						
Yilmaz Kaysin 2018 [58]	PT	VAS	−0.98	−2.36 to 0.40	//	⨁⨁◯◯ Low
	ST	VAS	−1.64	−2.98 to −0.31	SWD	
	IT	VAS	−2.10	−3.48 to −0.73	SWD	
**SWD** (+ Ultrasound + active exercises) vs. **Iontophoresis with acetic acid** (+ Ultrasound + active exercises)						
Jiménez-Garcia 2008 [59]	PT	VAS	−0.62	−2.01 to 0.77	//	⨁◯◯◯ Very low
**MWD vs. Subacromial corticosteroid injections**						
Rabini 2012 [77]	PT	VAS	5.50	−2.13 to 13.13	//	⨁◯◯◯ Very low
	IT	VAS	8.60	−1.41 to 18.61	//	
	LT	VAS	9.50	1.70 to 17.30	Subacromial corticosteroid injections	
**MWD vs. active exercises**						
Giombini 2006 [78]	PT	VAS	−2.90	−3.35 to −2.45	MWD	⨁◯◯◯ Very low
	IT	VAS	−3.70	−4.32 to −3.08	MWD	
**MWD vs. Ultrasound therapy**						
Giombini 2006 [78]	PT	VAS	−3.40	−3.99 to −2.81	MWD	⨁◯◯◯ Very low
	IT	VAS	−2.95	−3.54 to −2.36	MWD	
**MWD** (+ hot packs and active exercises) vs. **Sham MWD** (+ hot packs and active exercises)						
Akyol 2012 [76]	PT	VAS during activity	−0.60	−2.34 to 1.14	//	⨁◯◯◯ Very low
	ST	VAS during activity	−1.00	−2.68 to 0.68	//	
**CRET** (+ exercises) **vs. Sham CRET** (+ exercises)						
Avendaño-Coy 2022 [92]	PT	VAS at rest	0.15	−1.37, 1.67	//	⨁⨁◯◯ Low
	ST	VAS at rest	−0.05	−1.80, 1.70	//	
	IT	VAS at rest	0.20	−1.75, 2.15	//	

IT: Intermediate-Term follow up; LT: Long-Term follow up; MWD: Microwave Diathermy; PT: Post Treatment; ST: Short-Term follow up; SWD: Shortwave Diathermy; VAS: Visual Analogue Scale.

**Table 9:** Non-pooled data for improvement in function in STN.

Author Year	Assessment Time	Outcome Measure	MD Value	95% CI	Significantly in Favour of	GRADE
**SWD** (+ conservative treatment program) vs. **Sham SWD** (+ conservative treatment program)						
Yilmaz Kaysin 2018 [58]	PT	Constant-Murley total score	7.48	−0.56 to 15.52	//	⨁⨁◯◯ Low
	ST	Constant-Murley total score	10.48	2.65 to 18.32	SWD	
	IT	Constant-Murley total score	14.15	6.26 to 22.04	SWD	
**SWD** (+ Ultrasound + active exercises) **vs. Iontophoresis with acetic acid** (+ Ultrasound + active exercises)						
Jiménez-Garcia 2008 [59]	PT	Constant-Murley total score	−3.24	−13.27 to 6.79	//	⨁◯◯◯ Very low
**MWD vs. Subacromial corticosteroid injections**						
Rabini 2012 [77]	PT	QuickDASH	−3.90	−10.07 to 2.27	//	⨁◯◯◯ Very low
	IT	QuickDASH	6.10	−0.22 to 12.42	//	
	LT	QuickDASH	2.00	−6.34 to 10.34	//	
**MWD vs. active exercises**						
Giombini 2006 [78]	PT	Constant-Murley total score	16.90	13.54 to 20.26	MWD	⨁◯◯◯ Very low
	IT	Constant-Murley total score	18.73	14.28 to 23.18	MWD	
**MWD vs. Ultrasound therapy**						
Giombini 2006 [78]	PT	Constant-Murley total score	18.10	15.24 to 20.96	MWD	⨁◯◯◯ Very low
	IT	Constant-Murley total score	20.25	16.43 to 24.07	MWD	
**MWD** (+ hot packs and active exercises) vs. **Sham MWD** (+ hot packs and active exercises)						
Akyol 2012 [76]	PT	Shoulder Pain and Disability Index—Disability subscale	−2.35	−3.50 to −1.20	Sham MWD	⨁◯◯◯ Very low
	ST	Shoulder Pain and Disability Index—Disability subscale	−4.05	−5.23 to −2.87	Sham MWD	
**CRET** (+ exercises) **vs. Sham CRET** (+ exercises)						
Avendaño-Coy 2022 [92]	PT	QuickDASH	3.35	−8.98, 15.68	//	⨁⨁◯◯ Low
	ST	QuickDASH	−1.10	−13.88, 11.68	//	
	IT	QuickDASH	−1.40	−15.74, 12.94	//	

IT: Intermediate-Term follow up; LT: Long-Term follow up; MWD: Microwave Diathermy; PT: Post Treatment; ST: Short-Term follow up; SWD: Shortwave Diathermy.

**Table 10:** Non-pooled data for quality of life in STN.

Author Year	Assessment Time	Outcome Measure	MD Value	95% CI	Significantly in Favour of
**MWD** (+ hot packs and active exercises) vs. **Sham MWD** (+ hot packs and active exercises)					
Akyol 2012 [76]	PT	SF-36 general health subscale	−0.01	−0.09 to 0.07	//
	ST	SF-36 general health subscale	−0.05	−0.15 to 0.05	//
**CRET** (+ exercises) vs. **Sham CRET** (+ exercises)					
Avendaño-Coy 2022 [92]	PT	European Quality of Life—Five Dimensions	0.03	−0.07, 0.13	//
	ST	European Quality of Life—Five Dimensions	0.02	−0.11, 0.16	//
	IT	European Quality of Life—Five Dimensions	−0.02	−0.17, 0.13	//

CRET: Capacitive Resistive Electric Transfer; MWD: Microwave Diathermy; IT: Intermediate-Term follow up; PT: Post Treatment; ST: Short-Term follow up.

**Table 11:** Non-pooled data for pain relief in FS.

Author Year	Assessment Time	Outcome Measure	MD Value	95% CI	Significantly in Favour of	GRADE
**SWD + Hot packs** (+ pendulum, active stretching and exercises) vs. **Cyriax treatment** (+ pendulum and active stretching and exercises)						
Guler-Uysal 2008 [60]	PT	VAS (during motion)	12.10	0.03 to 24.17	Cyriax treatment	⨁◯◯◯ Very low

PT: Post Treatment; SWD: Shortwave Diathermy.

**Table 12:** Non-pooled data for improvement in function in FS.

Author Year	Assessment Time	Outcome Measure	MD Value	95% CI	Significantly in Favour of	GRADE
**SWD + Hot packs** (+ pendulum, active stretching and exercises) vs. **Cyriax treatment** (+ pendulum and active stretching and exercises)						
Guler-Uysal 2008 [60]	PT	VAS during motion	−21.60	−33.93 to −9.27	Cyriax treatment	⨁◯◯◯ Very low
**SWD** (+ stretching exercises) vs. **Hot packs** (+ stretching exercises)						
Leung 2008 [61]	PT	American Shoulder and Elbow Surgeons assessment form	11.30	−1.50 to 24.10	//	⨁◯◯◯ Very low
	ST	American Shoulder and Elbow Surgeons assessment form	13.50	−2.16 to 29.16	//	
**SWD + Stretching exercises vs. Stretching exercises**						
Leung 2008 [61]	PT	American Shoulder and Elbow Surgeons assessment form	21.70	9.47 to 33.93	SWD + Stretching exercises	⨁◯◯◯ Very low
	ST	American Shoulder and Elbow Surgeons assessment form	17.50	1.76 to 33.24	SWD + Stretching exercises	
**Diathermy [MWD or SWD] + Kaltenborn mobilization vs. Kaltenborn mobilization**						
Hammad 2019 [85]	ST	Shoulder pain and disability index	−51.80	−54.94 to −48.66	Diathermy	⨁◯◯◯ Very low

MWD: Microwave Diathermy; PT: Post Treatment; SWD: Shortwave Diathermy ST: Short-Term follow up.

**Table 13:** Non-pooled data for pain relief in LLT.

Author Year	Assessment Time	Outcome Measure	MD Value	95% CI	Significantly in Favour of	GRADE
**MWD vs. Ultrasound therapy**						
Giombini 2002 [80]	PT	VAS manual pressure pain	−2.10	−3.09 to −1.11	MWD	⨁◯◯◯ Very low
**Acupuncture + Ultrasound therapy + MWD vs. Extracorporeal shock wave therapy**						
Cheng 2018 [81]	PT	VAS	3.70	3.12 to 4.28	Extracorporeal shock wave therapy	⨁◯◯◯ Very low

MWD: Microwave Diathermy; PT: Post Treatment; VAS: Visual Analogue Scale.

**Table 14:** Non-pooled data for improvement in function in LLT.

Author Year	Assessment Time	Outcome Measure	MD Value	95% CI	Significantly in Favour of	GRADE
**Acupuncture + Ultrasound therapy + MWD vs. Extracorporeal shock wave therapy**						
Cheng 2018 [81]	PT	Extension muscle endurance	−0.06	−0.14 to 0.02	//	⨁◯◯◯ Very low

MWD: Microwave Diathermy; PT: Post Treatment.

**Table 15:** Non-pooled data for pain relief in NP.

Author Year	ASSESSMENT TIME	Outcome Measure	MD Value	95% CI	Significantly in Favour of	GRADE
**SWD + Education + Active exercises vs. Education + Active exercises**						
Dziedzic 2005 [64]	PT	Northwick Park Neck Pain Questionnaire	3.30	−0.94 to 7.54	//	⨁◯◯◯ Very low
	LT	Northwick Park Neck Pain Questionnaire	2.70	−2.06 to 7.46	//	
**SWD** (+ Education + Active exercises) vs. **Manual therapy** (+ Education + Active exercises)						
Dziedzic 2005 [64]	PT	Northwick Park Neck Pain Questionnaire	−0.70	−4.67 to 3.27	//	⨁◯◯◯ Very low
	LT	Northwick Park Neck Pain Questionnaire	−0.90	−5.78 to 3.98	//	
**MWD [continuous + pulsed]** (+ active exercises + TENS) vs. **Sham MWD** (+ active exercises + TENS)						
Ortega 2013 [82]	PT	VAS	1.54	−6.24 to 9.32	//	⨁⨁◯◯ Low
	LT	VAS	−1.41	−9.42 to 6.60	//	
**MWD continuous** (+ active exercises + TENS) vs. **MWD pulsed** (+ active exercises + TENS)						
Ortega 2013 [82]	PT	VAS	−3.40	−11.80 to 5.00	//	⨁⨁◯◯ Low
	LT	VAS	−1.60	−9.41 to 6.21	//	

LT: Long-Term follow up; MWD: Microwave Diathermy; PT: Post Treatment; SWD: Shortwave Diathermy; TENS: Trans-cutaneous Electrical Nerve Stimulation; VAS: Visual Analogue Scale.

**Table 16:** Non-pooled data for improvement in function in NP.

Author Year	Assessment Time	Outcome Measure	MD Value	95% CI	Significantly in Favour of	GRADE
**MWD [continuous + pulsed]** (+ active exercises + TENS) vs. **Sham MWD** (+ active exercises + TENS)						
Ortega 2013 [82]	PT	Neck disability Index	−1.55	−6.71 to 3.61	//	⨁⨁◯◯ Low
	LT	Neck disability Index	−2.06	−7.18 to 3.06	//	
**MWD continuous** (+ active exercises + TENS) vs. **MWD pulsed** (+ active exercises + TENS)						
Ortega 2013 [82]	PT	Neck disability Index	−0.10	−5.91 to 5.71	//	⨁⨁◯◯ Low
	LT	Neck disability Index	0.90	−4.74 to 6.54	//	

LT: Long-Term follow-up; MWD: Microwave Diathermy; PT: Post Treatment; TENS: Trans-cutaneous Electrical Nerve Stimulation.

**Table 17:** Non-pooled data for quality of life in NP.

Author Year	Assessment Time	Outcome Measure	MD Value	95% CI	Significantly in Favour of
**SWD + Education + Active exercises vs. Education + Active exercises**					
Dziedzic 2005 [64]	PT	SF-12 Mental component	−1.10	−3.64 to 1.44	//
	LT	SF-12 Mental component	0.50	−2.02 to 3.02	//
**SWD** (+ Education + Active exercises) **vs. Manual therapy** (+ Education + Active exercises)					
Dziedzic 2005 [64]	PT	SF-12 Mental component	−0.20	−2.72 to 2.32	//
	LT	SF-12 Mental component	0.60	−1.88 to 3.08	//
**MWD [continuous + pulsed]** (+ active exercises + TENS) vs. **Sham MWD** (+ active exercises + TENS)					
Ortega 2013 [82]	PT	SF-36 total score	1.64	−3.72 to 7.00	//
	LT	SF-36 total score	1.35	−3.99 to 6.69	//
**MWD continuous** (+ active exercises + TENS) vs. **MWD pulsed** (+ active exercises + TENS)					
Ortega 2013 [82]	PT	SF-36 total score	−4.00	−10.08 to 2.08	//
	LT	SF-36 total score	−3.90	−9.92 to 2.12	//

LT: Long-Term follow-up; MWD: Microwave Diathermy; PT: Post Treatment; SWD: Shortwave Diathermy; TENS: Trans-cutaneous Electrical Nerve Stimulation.

**Table 18:** Non-pooled data for pain relief in PFP.

Author Year	Assessment Time	Outcome Measure	MD Value	95% CI	Significantly in Favour of	GRADE
**Monopolar dielectric radiofrequency + Active exercises vs. Active exercises**						
Albornoz-Cabello 2020 [93]	PT	VAS worst pain (last 24 h)	−53.00	−59.22 to −46.78	Monopolar dielectric radiofrequency	⨁◯◯◯ Very low

PT: Post Treatment; VAS: Visual Analogue Scale.

**Table 19:** Non-pooled data for improvement in function in PFP.

Author Year	Assessment Time	Outcome Measure	MD Value	95% CI	Significantly in Favour of	GRADE
**Monopolar dielectric radiofrequency + Active exercises vs. Active exercises**						
Albornoz-Cabello 2020 [93]	PT	Lower Extremity Functionality Scale	22.00	15.45 to 28.55	Monopolar dielectric radiofrequency	⨁◯◯◯ Very low

PT: Post Treatment.

**Table 20:** Non-pooled data for pain relief in TMJ.

Author Year	Assessment Time	Outcome Measure	MD Value	95% CI	Significantly in Favour of	GRADE
**SWD vs. Ultrasound therapy**						
Talaat 1986 [67]	PT	Likert [0–3]	0.23	−0.15 to 0.61	//	⨁◯◯◯ Very low
**SWD vs. Tablet of methocarbamol + acetyl salicylic acid (Robaxisal)**						
Talaat 1986 [67]	PT	Likert [0–3]	−1.12	−1.49 to −0.75	SWD	⨁◯◯◯ Very low

PT: Post Treatment; SWD: Shortwave Diathermy.

**Table 21:** Non-pooled data for pain relief in DOMS.

Author Year	Assessment Time	Outcome Measure	MD Value	95% CI	Significantly in Favour of	GRADE
**CRET vs. Sham CRET**						
Visconti 2020 [94]	PT	NPRS	0.20	−0.94 to 1.34	//	⨁⨁◯◯ Low
**CRET vs. Massage**						
Visconti 2020 [94]	PT	NPRS	0.00	−1.21 to 1.21	//	⨁⨁◯◯ Low

CRET: Capacitive Resistive Electric Transfer; NPRS: Numeric Pain Rating Scale; PT: Post Treatment.

**Table 22:** Non-pooled data for improvement in function in DOMS.

Author Year	Assessment Time	Outcome Measure	MD Value	95% CI	Significantly in Favour of	GRADE
**CRET vs. No intervention**						
Nakamura 2022 [95]	PT	Maximum voluntary concentric contraction	49.70	20.25, 79.15	//	⨁◯◯◯ Very low

CRET: Capacitive Resistive Electric Transfer; PT: Post Treatment.

**Table 23:** Non-pooled data for pain relief in UNE.

Author Year	Assessment Time	Outcome Measure	MD Value	95% CI	Significantly in Favour of	GRADE
**SWD vs. Sham SWD**						
Badur 2020 [69]	PT	VAS	0.07	−1.30 to 1.44	//	⨁⨁◯◯ Low
	ST	VAS	−0.36	−1.66 to 0.94	//	
	IT	VAS	−0.37	−1.59 to 0.85	//	

IT: Intermediate-Term follow up; PT: Post Treatment; ST: Short-Term follow up; SWD: Shortwave Diathermy; VAS: Visual Analogue Scale.

**Table 24:** Non-pooled data for improvement in function in UNE.

Author Year	Assessment Time	Outcome Measure	MD Value	95% CI	Significantly in Favour of	GRADE
**SWD vs. Sham SWD**						
Badur 2020 [69]	PT	Quick-DASH	0.69	−9.69 to 11.07	//	⨁⨁◯◯ Low
	ST	Quick-DASH	3.70	−5.05 to 12.45	//	
	IT	Quick-DASH	−4.71	−14.13 to 4.71	//	

IT: Intermediate-Term follow up; PT: Post Treatment; ST: Short-Term follow-up; SWD: Shortwave Diathermy.

**Table 25:** Non-pooled data for QoL in UNE.

Author Year	Assessment Time	Outcome Measure	MD Value	95% CI	Significantly in Favour of
**SWD vs. Sham SWD**					
Badur 2020 [69]	PT	SF-36	0.98	−2.72 to 4.68	//
	ST	SF-36	1.04	−2.36 to 4.44	//
	IT	SF-36	1.03	−2.56 to 4.62	//

IT: Intermediate-Term follow-up; PT: Post Treatment; ST: Short-Term follow-up; SWD: Shortwave Diathermy.

**Table 26:** Non-pooled data for pain relief in LE.

Author Year	Assessment Time	Outcome Measure	MD Value	95% CI	Significantly in Favour of	GRADE
**SWD** + (transverse friction massage + stretching + strengthening + education) **vs. Sham SWD** + (transverse friction massage + stretching + strengthening + education)						
Babaei-Ghazani 2019 [70]	PT	VAS	−26.30	−32.60 to −20.00	SWD	⨁⨁◯◯ Low
	IT	VAS	−21.20	−26.11 to −16.29	SWD	

IT: Intermediate-Term follow-up; PT: Post Treatment; SWD: Shortwave Diathermy; VAS: Visual Analogue Scale.

**Table 27:** Non-pooled data for improvement in function in LE.

Author Year	Assessment Time	Outcome Measure	MD Value	95% CI	Significantly in Favour of	GRADE
**SWD** + (transverse friction massage + stretching + strengthening + education) **vs. Sham SWD** + (transverse friction massage + stretching + strengthening + education)						
Babaei-Ghazani 2019 [70]	PT	Quick-DASH	−21.20	−28.52 to −13.88	SWD	⨁⨁◯◯ Low
	IT	Quick-DASH	−17.20	−23.39 to −11.01	SWD	

IT: Intermediate-Term follow-up; PT: Post Treatment; SWD: Shortwave Diathermy.

**Table 28:** Non-pooled data of pain relief in LAMI.

Author Year	Assessment Time	Outcome Measure	MD Value	95% CI	Significantly in Favour of	GRADE
**MWD vs. Ultrasound therapy**						
Giombini 2001 [83]	PT	VAS pain pressure and active resisted contraction of the muscle involved	−2.20	−2.90 to −1.50	MWD	⨁◯◯◯ Very low

MWD: Microwave diathermy; PT: Post Treatment; VAS: Visual Analogue Scale.

**Table 29:** Non-pooled data for pain relief in TKR.

Author Year	Assessment Time	Outcome Measure	MD Value	95% CI	Significantly in Favour of	GRADE
**CRET + usual physiotherapy vs. Usual physiotherapy**						
García-Marín 2021 [96]	PT	VAS	−1.21	−2.93 to 0.51	//	⨁⨁◯◯ Low
**CRET + usual physiotherapy vs. Sham CRET + usual physiotherapy**						
García-Marín 2021 [96]	PT	VAS	−1.11	−2.46 to 0.24	//	⨁⨁◯◯ Low

CRET: Capacitive Resistive Electric Transfer; PT: Post Treatment; VAS: Visual Analogue Scale.

**Table 30:** Non-pooled data for improvement in function in TKR.

Author Year	Assessment Time	Outcome Measure	MD Value	95% CI	Significantly in Favour of	GRADE
**CRET + usual physiotherapy vs. Usual physiotherapy**						
García-Marín 2021 [96]	PT	WOMAC total score	−0.04	−11.95 to 11.87	//	⨁⨁◯◯ Low
**CRET + usual physiotherapy vs. Sham CRET + usual physiotherapy**						
García-Marín 2021 [96]	PT	WOMAC total score	−1.16	−14.07 to 11.75	//	⨁⨁◯◯ Low

CRET: Capacitive Resistive Electric Transfer; PT: Post Treatment; VAS: Visual Analogue Scale.

**Table 31:** Non-pooled data for Qol improvement in TKR.

Author Year	Assessment Time	Outcome Measure	MD Value	95% CI	Significantly in Favour of
**CRET + usual physiotherapy vs. Usual physiotherapy**					
García-Marín 2021 [96]	PT	SF-12 mental component	−4.32	−9.88 to 1.24	//
**CRET + usual physiotherapy vs. Sham CRET + usual physiotherapy**					
García-Marín 2021 [96]	PT	SF-12 mental component	4.92	−1.42 to 11.26	//

CRET: Capacitive Resistive Electric Transfer; PT: Post Treatment; VAS: Visual Analogue Scale.

## Data Availability

The data presented in this study are available on request from the corresponding author.

## Supplementary Materials

The following supporting information can be downloaded at: https://www.mdpi.com/article/10.3390/jcm12123956/s1.
