# Supplementary material for: The Efficacy of Electromagnetic Diathermy for the Treatment of Musculoskeletal Disorders: A Systematic Review with Meta-Analysis
Source: J Clin Med. 2023 Jun 9;12(12):3956. doi: 10.3390/jcm12123956 (PMC10299724; doi:10.3390/jcm12123956)
Supplement: Supplementary file 1 [file jcm-12-03956-s001.zip › File S1.pdf]

Supplementary Materials File S1 – Outcome measure hierarchy.

Osteoarthritis	Pain relief	Function	Quality of life
	1. Visual Analogue Scale<br>2. Numerical Pain Rating Scale<br>3. WOMAC (pain)<br>4. Likert scale	1. WOMAC (function)<br>2. WOMAC (total)<br>3. Lequesne Index<br>4. Lysholm Knee Scoring Scale<br>5. Stair Climbing Test<br>6. Walk Test (10/15/x meter)<br>7. Timed Up and Go Test<br>8. Strength measures<br>9. Range of Motion	1. SF-36 (overall scale)<br>2. SF-36 (general health)<br>3. SF-36 (physical functioning)<br>4. KOOS (QoL subscale)<br>5. General Health Questionnaire
**Low Back Pain**	1. Visual Analogue Scale<br>2. Numerical Pain Rating Scale<br>3. Likert scale<br>4. Lattinen Test score	1. Oswestry Disability Index<br>2. Roland Morris Disability Questionnaire	1. SF-36 (overall scale)<br>2. SF-36 (general health)<br>3. SF-36 (physical functioning)
**Shoulder tendinopathies**	1. Visual Analogue Scale<br>2. Numerical Pain Rating Scale<br>3. Likert scale	1. QuickDASH score<br>2. Constant-Murley score (ADL)<br>3. Contant-Murley score (general)<br>4. Shoulder Pain and Disability Index (disability scale)<br>5. Shoulder Pain and Disability Index (total score)<br>6. Shoulder Disability Questionnaire<br>7. Strength measure<br>8. Range of Motion (active)<br>9. Range of Motion (passive)	1. SF-36 (overall scale)<br>2. SF-36 (general health)<br>3. SF-36 (physical functioning)
**Frozen shoulder**	1. Visual Analogue Scale<br>2. Numerical Pain Rating Scale<br>3. Likert scale	1. QuickDASH score<br>2. Constant-Murley score (ADL)<br>3. Contant-Murley score (general)<br>4. Shoulder Pain and Disability Index (disability scale)	1. SF-36 (overall scale)<br>2. SF-36 (general health)<br>3. SF-36 (physical functioning)

		5. Shoulder Pain and Disability Index (total score)<br>6. Shoulder Disability Questionnaire<br>7. Range of Motion (passive)<br>8. Range of Motion (active)<br>9. Strength measure	

ADL: Activity of Daily Living ; DASH: Disability of the Arm, Shoulder and Hand score ; KOOS: Knee Injury and Osteoarthritis Outcome Score ; SF-36: La Short Form Health Survey 36 ; WOMAC: Western Ontario and McMaster University Osteoarthritis Index ; QoL: Quality of Life.
