# Supplementary material for: The Efficacy of Electromagnetic Diathermy for the Treatment of Musculoskeletal Disorders: A Systematic Review with Meta-Analysis
Source: J Clin Med. 2023 Jun 9;12(12):3956. doi: 10.3390/jcm12123956 (PMC10299724; doi:10.3390/jcm12123956)
Supplement: Supplementary file 1 [file jcm-12-03956-s001.zip › File S2.pdf]

	Random sequence generation (selection bias)	Allocation concealment (selection bias)	Blinding of participants and personnel (performance bias)	Blinding of outcome assessment (detection bias)	Incomplete outcome data (attrition bias)	Selective reporting (reporting bias)	Other bias
Ahmed 2009	?	?	?	?	−	?	?
Akyol 2010	?	+	−	+	+	?	?
Akyol 2012	?	+	?	+	+	?	?
Albornoz-Cabello 2020	+	+	−	+	+	−	?
Alcidi 2007	?	?	−	−	+	?	?
Ansari 2022	+	?	−	+	+	+	?
Atamaz 2006	?	?	−	?	+	?	?
Atamaz 2012	?	+	+	+	+	?	?
Avendaño-Coy 2022	+	?	+	+	+	+	?
Babaei-Ghazani 2019	?	+	+	+	+	+	?
Badur 2020	+	+	+	+	+	+	?
Bazael 2010	+	?	?	+	+	?	?
Boyaci 2013	?	+	−	?	+	?	?
Boyaci 2014	?	+	?	+	+	?	?
Callaghan 2005	?	+	+	+	+	?	?
Cantarini 2006	?	?	−	+	+	?	?
Cetin 2008	?	?	−	+	−	?	?
Chamberlain 1982	?	?	−	+	−	?	?
Cheng 2018	+	?	−	?	+	?	?
Clarke 1974	?	?	−	+	?	?	?
Coccetta 2018	+	+	?	+	+	+	?
Durmus 2014	?	+	−	?	+	?	?
Dziedzic 2005	+	+	−	?	+	?	?
Farrell 1982	?	?	−	?	?	?	?
Frasca 2011	+	?	+	+	+	?	?
Fukuda 2008	?	?	?	+	+	?	?
Fukuda 2011	?	+	?	+	?	+	?
García-Marín 2021	+	+	−	+	+	+	?
Georgoudis 2017	+	?	−	+	+	−	?
Gibson 1985	?	?	−	+	+	?	?
Giombini 2001	+	+	−	?	+	?	?
Giombini 2002	+	?	−	+	+	?	?
Giombini 2006	+	?	−	+	+	?	?
Giombini 2010	+	+	+	?	+	?	?
Gomes 2020	?	+	−	+	+	+	?
Gray 1995	+	?	−	+	+	?	?
Guler-Uysal 2004	?	?	−	?	+	?	?
Hammad 2019	+	+	−	?	+	?	?
Igatpurikar 2013	?	?	−	?	+	?	?
Incebiyik 2015	?	+	+	+	+	?	?
Işik 2020	−	−	+	?	?	?	?
Jia 2022	+	+	?	?	+	+	?
Jiménez-Garcia 2008	?	?	?	?	−	?	?
Klaber Moffett 1996	+	+	?	?	−	−	?
Kumar 2009	+	+	−	−	+	?	?
Kumar 2009a	+	+	−	?	+	?	?
Kumaran 2019	+	+	−	?	+	?	?
Leung 2008	+	?	−	+	+	?	?
Livesley 1992	?	?	−	?	−	?	?
Nakamura 2022	−	?	−	−	+	?	?
Notarnicola 2017	?	?	−	?	+	?	?
Ortega 2013	+	?	+	+	+	+	?
Ovanessian 2008	+	?	?	+	−	?	?
Pasila 1978	+	?	?	?	−	?	?
Rabini 2012	+	+	−	?	+	?	?
Rabini 2012a	+	+	−	+	+	?	?
Rattanachaiyanont 2008	+	+	+	+	−	−	?
Shakoor 2008	+	?	+	−	−	−	?
Taalat 1986	?	?	−	−	?	?	?
Terzi 2017	?	?	−	?	?	?	?
Teslim 2013	+	+	?	?	+	?	?
Tüzün 2003	?	?	?	?	+	?	?
Verma 2012	?	?	?	−	?	?	?
Visconti 2020	+	?	+	+	+	+	?
Wachi 2022	+	?	−	+	+	+	?
Wright 1964	?	?	−	?	−	?	?
Yilmaz 2018	+	+	+	+	?	?	?
Zati 2018	+	?	−	?	+	?	?
