# Supplementary material for: The Efficacy of Electromagnetic Diathermy for the Treatment of Musculoskeletal Disorders: A Systematic Review with Meta-Analysis
Source: J Clin Med. 2023 Jun 9;12(12):3956. doi: 10.3390/jcm12123956 (PMC10299724; doi:10.3390/jcm12123956)
Supplement: Supplementary file 1 [file jcm-12-03956-s001.zip › File S3.pdf]

AUTHOR & YEAR	PATHOLOGY	TYPE OF DIATHERMY	DEVICE	FREQUENCY
Akyol 2010	Knee and hip osteoarthritis	SWD	Curapuls 491	27.12 Mhz
Atamaz 2006	Knee and hip osteoarthritis	SWD	NR	NR
Atamaz 2012	Knee and hip osteoarthritis	SWD	Intelect shortwave Model n. 3812	NR
Bezalel 2010	Knee and hip osteoarthritis	SWD	Curapuls 670 (Enraf-Nonius)	300 Hz

AUTHOR & YEAR	PATHOLOGY	TYPE OF DIATHERMY	DEVICE	FREQUENCY
Boyaci 2013	Knee and hip osteoarthritis	SWD	Curapuls 970 (Enraf-Nonius)	27.12 MHz
Callaghan 2005	Knee and hip osteoarthritis	SWD	EMS Megapulse (EMS Ltd, UK)	27 MHz
Cantarini 2006	Knee and hip osteoarthritis	SWD	SIMENS Ultraterm 642 E	NR
Cetin 2008	Knee and hip osteoarthritis	SWD	Curapuls 419 (Enraf-NoniusB Delftechpark 39)	27.12 MHz
Chamberlain 1982	Knee and hip osteoarthritis	SWD	NR	NR
Clarke 1974	Knee and hip osteoarthritis	SWD	NR	NR
Fukuda 2011	Knee and hip osteoarthritis	SWD	Diatermed II	27.12 MHz
Fukuda 2008	Knee and hip osteoarthritis	SWD	Diatermed II	27.12 MHz

AUTHOR & YEAR	PATHOLOGY	TYPE OF DIATHERMY	DEVICE	FREQUENCY
Gomes 2020	Knee and hip osteoarthritis	SWD	Ibramed (Amparo, SP, Brasil)	27.12 Mhz
Işik 2020	Knee and hip osteoarthritis	SWD	Curapuls 970 (Enraf-Nonius)	27.12MHz
Jia 2022	Knee and hip osteoarthritis	SWD	Curapulse 970 (Enraf–Nonius)	27.12 Mhz
Klaber Moffett 1996/1994	Knee and hip osteoarthritis	SWD	Ukramed IIS 601	NR
Ovanessian 2008	Knee and hip osteoarthritis	SWD	Diatermed II	27.12MHz
Rattanachaiyanont 2008	Knee and hip osteoarthritis in peri-/post-menopausal women	SWD	ULTRAMED (Bosch) model 11s601	27.12MHz

AUTHOR & YEAR	PATHOLOGY	TYPE OF DIATHERMY	DEVICE	FREQUENCY
Terzi 2017	Knee and hip osteoarthritis	SWD	NR	NR
Teslim 2013	Knee and hip osteoarthritis	SWD	Curapuls 967	NR
Tüzün 2003	Knee and hip osteoarthritis	SWD	Curapuls 419 (Enraf-Nonius)	110 Hz and 20 Hz
Wright 1964	Knee and hip osteoarthritis	SWD	NR	NR

AUTHOR & YEAR	PATHOLOGY	TYPE OF DIATHERMY	DEVICE	FREQUENCY
Giombini 2010	Knee and hip osteoarthritis	MWD	‘’ALBA Hyperthermia System’’ (RESTEK SRL ITALY)	433.92 MHz
Rabini 2012	Knee and hip osteoarthritis	MWD	Smarterapia Sigma Hyperthermia System (Easytech srl, Borgo San Lorenzo, Florence, Italy)	434 MHz
Alcidi 2007	Knee and hip osteoarthritis	LPRER	NR (own built)	500 KHz
Cocetta 2018	Knee and hip osteoarthritis	CRET	Tecar Unibell HCR 902 device (Unibell, Calco, Lecco, Italy)	485 KHz
Kumaran 2019	Knee and hip osteoarthritis	CRET	Indiba Activ 902	448 KHz
Ahmed 2009	Low back pain	SWD	NR	NR
Ansari 2022	Low back pain	SWD	NR	27.12 Mhz
Gibson 1985	Low back pain	SWD	NR	NR

AUTHOR & YEAR	PATHOLOGY	TYPE OF DIATHERMY	DEVICE	FREQUENCY
Igatpurkar 2013	Low back pain	SWD	NR	NR
Kumar 2009	Low back pain	SWD	Medichem Electronics	NR
Kumar 2009a	Low back pain	SWD	Medichem Electronics	NR
Shakoor 2008	Low back pain	SWD	NR	27.33 MHz
Durmus 2014	Low back pain	MWD	Curadar 409 (Enraf–Nonius, The Netherlands)	2.450 MHz

AUTHOR & YEAR	PATHOLOGY	TYPE OF DIATHERMY	DEVICE	FREQUENCY
Farrell 1982	Low back pain	MWD	NR	NR
Notarnicola 2017	Low back pain	CRET	Doctor Tecar Therapy™, Mectronic Medical SRL, Bergamo, Italy	Betweeen 0.45 and 0.60 MHz
Wachi 2022	Low back pain	CRET	Physio Radio Stim Pro (SAKAI Medical Co., Ltd., Tokyo, Japan)	500 kHz
Zati 2018	Low back pain	CRET	NR	450-1000 kHz
Jiménez-Garcia 2008	Shoulder tendinopathies	SWD	Elios-uniphy-phyaction performa	27 MHz
Yilmaz 2018	Shoulder tendinopathies	SWD	ITO SW-180 device	27.12 Mhz

AUTHOR & YEAR	PATHOLOGY	TYPE OF DIATHERMY	DEVICE	FREQUENCY
Akyol 2012	Shoulder tendinopathies	MWD	Curadar 409 (Enraf–Nonius, The Nederland)	2.450 MHz
Giombini 2006	Shoulder tendinopathies in athletes	MWD	‘’ALBA Hyperthermia System’’ (RESTEK SRL ITALY)	433.92 MHz
Rabini 2012	Shoulder tendinopathies	MWD	Smarterapia Sigma Hyperthermia System (Easytech srl, Borgo San Lorenzo, Florence, Italy)	434 MHz
Avendaño-Coy 2022	Shoulder tendinopathies	CRET	INDIBA Activ CT9 device (INDIBA S.A., Spain)	448 Khz
Guler-Uysal 2004	Frozen shoulder	SWD	KSF Model equipment ITO (Tokyo-Japan)	27.12 Mhz
Leung 2008	Frozen shoulder	SWD	Curapuls 419 (Enraf-Nonius)	27.12 Mhz
Hammad 2019	Frozen shoulder	SWD/MWD	NR	NR

AUTHOR & YEAR	PATHOLOGY	TYPE OF DIATHERMY	DEVICE	FREQUENCY
Boyaci 2014	Carpal tunnel syndrome	SWD	Curapuls 970 (Enraf-Nonius)	NR
Incebiyik 2015	Carpal tunnel syndrome	SWD	Curapuls 970 (Enraf-Nonius)	27 MHz
Frasca 2011	Carpal tunnel syndrome	MWD	Smarterapia Sigma Hyperthermia System (Easytech srl, Borgo San Lorenzo, Florence, Italy)	434 MHz
Cheng 2018	Lower limb tendinopathies in athletes	MWD	Fysiomed (Edegem, Belgium)	NR
Giombini 2002	Lower limb tendinopathies in athletes	MWD	‘‘ALBA Hyperthermia System’’ (RESTEK SRL ITALY)	434 MHz
Dziedzic 2005	Neck pain	SWD	NR	NR
Ortega 2013	Neck pain	MWD	Enraf–Nonius Radarmed	NR
Verma 2012	Patellofemoral pain	SWD	NR	27.12 Mhz

AUTHOR & YEAR	PATHOLOGY	TYPE OF DIATHERMY	DEVICE	FREQUENCY
Albornoz-Cabello 2020	Patellofemoral pain	MDR	ABD Modular (Biotronic Advance Develops, Spain)	840 Khz
Gray 1995	Temporomandibular pain dysfunction syndrome	SWD.	NR	NR
Talaat 1986	Temporomandibular pain dysfunction syndrome	SWD	Ultratherma 708-Siemens	NR
Nakamura 2022	DOMS in healthy subjects	CRET	Indiba Active Pro Recovery HCR904	448 Khz
Visconti 2020	DOMS in athletes	CRET	Red Coral, Tecnosix, Sixtus (Italy)	750 Khz
Livesley 1992	Fractures of the neck of the humerus	SWD	Curapuls	NR
Babaei-Ghazani 2019	Lateral epicondilitis	SWD	Shortwave Unit SW 400s, ITO CO (Germany)	27.12 Mhz
Badur 2020	Ulnar nerve entrapment	SWD	NR	27.12 Mhz
Pasila 1978	Ankle or foot sprain	SWD	NR	NR
Giombini 2001	Acute muscle injury in athletes	MWD	‘‘ALBA Hyperthermia System’’ (RESTEK SRL ITALY)	433.92 MHz

AUTHOR & YEAR	PATHOLOGY	TYPE OF DIATHERMY	DEVICE	FREQUENCY
Georgoudis 2017	Tension-type headache	MWD	NR	NR
García-Marín 2021	Total Knee Replacement pain	CRET	Biotronic Advance Develops (Granada, Spain)	840 Khz

AUTHOR & YEAR	INTERVENTION I	INTERVENTION II	INTERVENTION III	INTERVENTION IV	INTERVENTION V
Akyol 2010	SWD + active exercises. SWD: 40 min, 12 sessions, 3 days a week for 4 weeks; active exercises: 12 sessions, 3 days a week for 4 weeks.				
Atamaz 2006	SWD + infrared therapy + interferential therapy. SWD: 20 min, 15 sessions, 5 days a week for 3 weeks; infrared therapy: 20 min, 15 sessions, 5 days a week for 3 weeks; interferential therapy: 20 min, 15 sessions, 5 days a week for 3 weeks.				
Atamaz 2012	SWD + active exercises + home based exercises + education. SWD: 15 sessions, 5 days a week for 3 weeks; active exercises: 9 sessions, 3 days a week for 3 weeks; home based exercises: NR; education: 60 min, 1 sessions.				
Bezalel 2010	SWD. 20 min, 6 sessions.				

AUTHOR & YEAR	INTERVENTION I	INTERVENTION II	INTERVENTION III	INTERVENTION IV	INTERVENTION V
Boyaci 2013	SWD. 20 min, 10 sessions, 5 days a week for 2 weeks.				
Callaghan 2005	SWD high dose. 20 min, 6 sessions, 3 days a week for 2 weeks.	SWD low dose. 20 min, 6 sessions, 3 days a week for 2 weeks.			
Cantarini 2006	SWD. 15 min, 10 sessions, 3 days a week for 3 weeks.				
Cetin 2008	SWD + hot pack + active exercises . SWD: 15 min, 24 sessions, 3 days a week for 8 weeks; hot packs and exercise: 24 sessions, 3 days a week for 8 weeks.				
Chamberlain 1982	SWD. 12 sessions, 3 days a week for 4 weeks.				
Clarke 1974	SWD. 9 sessions, 3 days a week for 3 weeks.				
Fukuda 2011	SWD high dose. 38 min, 9 sessions, 3 days a week for 3 weeks.	SWD low dose. 19 min, 9 sessions, 3 days a week for 3 weeks.			
Fukuda 2008	SWD high dose. 38 min, 9 sessions, 3 days a week for 3 weeks.	SWD low dose. 19 min, 9 sessions, 3 days a week for 3 weeks.			

AUTHOR & YEAR	INTERVENTION I	INTERVENTION II	INTERVENTION III	INTERVENTION IV	INTERVENTION V
Gomes 2020	SWD + active exercises. SWD: 20 min, 24 sessions, 3 days a week for 8 weeks; active exercises: 90 min, 24 sessions, 3 days a week for 8 weeks.				
Işik 2020	SWD + injection + home based active exercises. SWD and injection: 20 min, 3 sessions, once a week for 3 weeks; home-based exercise: 40 min, 36 sessions, 6 days a week for 6 weeks.				
Jia 2022	SWD. 20 min, 12 sessions, once a day.				
Klaber Moffett 1996/1994	SWD. 15 min, 9 sessions, 3 days a week for 3 weeks.				
Ovanessian 2008	SWD 33kj. 38 min, 9 sessions, 3 days a week for 3 weeks.	SWD 17 kj. 19 min, 9 sessions, 3 days a week for 3 weeks.			
Rattanachaiyanont 2008	SWD + active exercises + education. SWD: 20 min, 9 sessions, 3 days a week for 3 weeks. Active exercise and education: NR.				

AUTHOR & YEAR	INTERVENTION I	INTERVENTION II	INTERVENTION III	INTERVENTION IV	INTERVENTION V
Terzi 2017	SWD + education + active exercises + TENS + hot pack. SWD: 15 min, 10 sessions, 5 days a week for 2 weeks; education: NR; active exercises: 84 at home sessions, 14 times a week (twice a day) for 6 weeks; TENS: 20 min, 10 sessions, 5 days a week for 2 weeks; hot packs: 20 min, 10 sessions, 5 days a week for 2 weeks.				
Teslim 2013	SWD continuos + active exercises + massage with diclofenac. SWD continuous: 20 min, 8 sessions, 2 days a week for 4 weeks; active exercise: 15 min, 8 sessions, 2 days a week for 4 weeks; massage with diclofenac: NR.	SWD pulsed + active exercises + massage with diclofenac. SWD pulsed: 20 min, 8 sessions, 2 days a week for 4 weeks; active exercise: 15 min, 8 sessions, 2 days a week for 4 weeks; massage with diclofenac: NR.			
Tüzün 2003	SWD high power + ultrasound + active exercises. SWD high power: 15 min, 10 sessions, 5 days a week for 2 weeks; ultrasound: 5 min, 10 sessions, 5 days a week for 2 weeks; active exercises: 10 min, 10 sessions, 5 days a week for 2 weeks.	SWD low power + ultrasound + active exercises. SWD low power: 15 min, 10 sessions, 5 days a week for 2 weeks; ultrasound: 5 min, 10 sessions, 5 days a week for 2 weeks; active exercises: 10 min, 10 sessions, 5 days a week for 2 weeks.			
Wright 1964	SWD. 20 minutes, 18 sessions, 3 days a week for 6 weeks.				

AUTHOR & YEAR	INTERVENTION I	INTERVENTION II	INTERVENTION III	INTERVENTION IV	INTERVENTION V
Giombini 2010	MWD. 30 min, 12 sessions, 3 days a week for 4 weeks.				
Rabini 2012	MWD. 30 min, 12 sessions, 3 days a week for 4 weeks.				
Alcidi 2007	LPRER. 20 min, 5 sessions, 5 days for 1 weeks.				
Cocetta 2018	CRET. 20 min (5 min of capacitive, 10 min of resistive, 5 min of capacitive), 6 sessions, 3 days a week for 2 weeks.				
Kumaran 2019	CRET + home based active exercises + education. CRET: 15 minutes (5 min of capicitive and 10 min of resistive), 8 sessions, 2 days a week for 4 weeks; home based exercise + education: 3 sessions in 4 weeks.				
Ahmed 2009	SWD + drug + active exercises + education. SWD: 15 min, 18 sessions, 3 days a week for 6 weeks; drug: 7 days a week; active exercise: NR; education: NR.				
Ansari 2022	SWD. 20 min, 10 sessions, on alternate days for 3 weeks.				
Gibson 1985	SWD. 12 sessions, 3 days a week for 4 weeks.				

AUTHOR & YEAR	INTERVENTION I	INTERVENTION II	INTERVENTION III	INTERVENTION IV	INTERVENTION V
Igatpurkar 2013	SWD + traction + active exercises. SWD: NR; traction: 15 min; active exercises: NR.				
Kumar 2009	SWD + ultrasound + active exercises. SWD: 15 min, 18 sessions; ultrasound: 8 min, 18 sessions; active exercises: NR.				
Kumar 2009a	SWD + ultrasound + active exercises. SWD: 15 min, 20 sessions; ultrasound: 5 min, 20 sessions; active exercises: 20 sessions.				
Shakoor 2008	SWD + drug + active exercises + education. SWD: 15 min, 18 sessions, 3 days a week for 6 weeks; drug: twice a day 7 days a week; active exercise: 84 sessions, twice a day 7 days a week for 6 weeks; education: NR.				
Durmus 2014	MWD + active exercises. MWD: 20 min, 18 sessions, 3 days a week for 6 weeks; active exercises: 60 min, 18 sessions, 3 days a week for 6 weeks.				

AUTHOR & YEAR	INTERVENTION I	INTERVENTION II	INTERVENTION III	INTERVENTION IV	INTERVENTION V
Farrell 1982	MWD + active exercises + education. MWD: 15 min, 9 sessions, 3 days a week for 3 weeks; active exercises: 9 sessions, 3 days a week for 3 weeks (also 3 or 4 days of home-based exercise); education: 9 sessions, 3 days a week for 3 weeks.				
Notarnicola 2017	CRET. 20 min (10 min of capacitive, 10 min of resistive), 10 sessions, 5 days a week for 2 weeks.				
Wachi 2022	CRET. 15 min (5 min of capacitive, 10 min of resistive).				
Zati 2018	Deep Heathing Therapy. 20 min, 10 sessions in 2 weeks.	Superficial Heathing Therapy. 20 min, 10 sessions in 2 weeks.			
Jiménez-Garcia 2008	SWD + ultrasound + active exercises. SWD: 15 sessions, 5 days a week for 3 weeks; ultrasound: 5 min, 15 sessions, 5 days a week for 3 weeks; active exercise: 15 sessions, 5 days a week for 3 weeks.				
Yilmaz 2018	SWD + active exercises + cold pack + drug. SWD: 20 min, 10 sessions, 5 days a week for 2 weeks; active exerise: NR; cold pack: 20 min, 3 times a day; drug: 2 weeks.	SWD + active exercises + cold pack + drug. SWD: 20 min, 10 sessions, 5 days a week for 2 weeks; active exerise: NR; cold pack: 20 min, 3 times a day; drug: 2 weeks.			

AUTHOR & YEAR	INTERVENTION I	INTERVENTION II	INTERVENTION III	INTERVENTION IV	INTERVENTION V
Akyol 2012	MWD + hot pack + active exercises. MWD: 20 min, 15 sessions, 5 days a week for 3 weeks; hot packs: 20 min, 15 sessions, 5 days a week for 3 weeks; active exercises: 15 min, 15 sessions, 5 days a week for 3 weeks.				
Giombini 2006	MWD. 30 min, 12 sessions, 3 days a week for 4 weeks.				
Rabini 2012	MWD. 30 min, 12 sessions, 3 days a week for 4 weeks.				
Avendaño-Coy 2022	CRET thermal + exercises. CRET thermal: 15 min, 9 sessions, 3 days a week for 3 weeks; exercises: 15 sessions, 5 days a week for 3 weeks.	CRET subthermal + exercises. CRET subthermal: 15 min, 9 sessions, 3 days a week for 3 weeks; exercises: 15 sessions, 5 days a week for 3 weeks.			
Guler-Uysal 2004	SWD + hot pack + active exercises. SWD: 20 min; hot pack: 20 min; active exercises: NR.				
Leung 2008	SWD + stretching. SWD: 20 min; stretching: 12 sessions, 3 days a week for 4 weeks.				
Hammad 2019	MWD + SWD + hot pack + mobilization. MWD/SWD/hot pack: 15 min, 9 sessions, 3 days a week for 3 weeks; mobilization: 9 sessions, 3 days a week for 3 weeks.				

AUTHOR & YEAR	INTERVENTION I	INTERVENTION II	INTERVENTION III	INTERVENTION IV	INTERVENTION V
Boyaci 2014	SWD continuos. 20 min, 15 sessions, 5 days a week for 3 weeks.	SWD pulsed. 20 min, 15 sessions, 5 days a week for 3 weeks.			
Incebiyik 2015	SWD + hot pack + nerve gliding exercises. SWD: 15 min, 15 sessions, 5 days a week for 3 weeks; hot pack: 15 min, 15 sessions, 5 days a week for 3 weeks; nerve gliding exercises: 15 sessions, 5 days a week for 3 weeks.				
Frasca 2011	MWD. 20 min, 6 sessions, 2 days a week for 3 weeks.				
Cheng 2018	MWD + acupuncture + ultrasound. MWD: 20 min, 16 sessions, once a week for 16 weeks; acupuncture: NR; ultrasound: 8 min, 16 sessions, once a week for 16 week.				
Giombini 2002	MWD. 30 min, 12 sessions, 3 days a week for 4 weeks.				
Dziedzic 2005	SWD + education. 20 min, 8 sessions, 6 weeks.				
Ortega 2013	MWD continuos + active exercises + TENS. 15 sessions, 5 days a week for 3 weeks.	MWD pulsed + active exercises + TENS. 15 sessions, 5 days a week for 3 weeks.			
Verma 2012	SWD + active exercises. SWD:10 min, 2 weeks; active exercise NR.				

AUTHOR & YEAR	INTERVENTION I	INTERVENTION II	INTERVENTION III	INTERVENTION IV	INTERVENTION V
Albornoz-Cabello 2020	MDR + active exercises. MDR: NR; active exercises: 10 min, 10 sessions, 5/3/2 days a weeks progressively for 3 weeks.				
Gray 1995	SWD. 10 min, 12 sessions, 3 days a week for 4 weeks.				
Talaat 1986	SWD. 20 min, 14 sessions, 7 days a week for 2 weeks.				
Nakamura 2022	CRET. 30 min, 1 session.				
Visconti 2020	CRET. 10 min, 1 session.				
Livesley 1992	SWD + standard physiotherapy. SWD: 30 min, 10 sessions; standard physiotherapy: NR.				
Babaei-Ghazani 2019	SWD + massage + stretching + active exercises + education. SWD: 15 min, 10 sessions; massage + stretching + active exercises + education: NR.				
Badur 2020	SWD. 20 min, 10 sessions, 5 days a week for 2 weeks.				
Pasila 1978	SWD diapulse. 20 min, 3 sessions.	SWD curapulse. 20 min, 3 sessions.			
Giombini 2001	MWD. 30 min, 9 sessions, 3 days a week for 3 weeks.				

AUTHOR & YEAR	INTERVENTION I	INTERVENTION II	INTERVENTION III	INTERVENTION IV	INTERVENTION V
Georgoudis 2017	MWD + acupuncture + stretching + myofascial release. MWD: 10 min, 10 sessions; acupuncture: 20 min, 10 sessions; stretching: NR, 2-3 times daily; myofascial release: 15 min, 10 sessions.				
García-Marín 2021	LWD + active exercises. LWD: 12 min, 10 sessions, 5 days a week for 2 weeks; active exercises: 30 min, 10 sessions, 5 days a week for 2 weeks.				

AUTHOR & YEAR	CONTROL I	CONTROL II	CONTROL III	CONTROL IV	CONTROL V
Akyol 2010	Active exercise. 12 sessions, 3 days a week for 4 weeks.				
Atamaz 2006	Hyaluronic acid injection (NaHA). 4 sessions, 2 days a week for the first week (at day 0 and 7), 1 day a week for the second week (at day 14) and at 6 months.	Hyaluronic acid injection (Hylan). 4 sessions, 2 days a week for the first week (at day 0 and 7), 1 day a week for the second week (at day 14) and at 6 months.			
Atamaz 2012	Sham TENS + active exercises + home based exercises + education. Sham TENS: 20 min, 15 sessions, 5 days a week for 3 weeks; active exercises: 9 sessions, 3 days a week for 3 weeks; home based exercises: NR; education: 60 min, 1 sessions.	TENS + active exercises + home based exercises + education. TENS: 20 min, 15 sessions, 5 days a week for 3 weeks; active exercises: 9 sessions, 3 days a week for 3 weeks; home based exercises: NR; education: 60 min, 1 sessions.	Sham interferential currents + active exercises + home based exercises + education. Sham interferential currents: 20 min, 15 sessions, 5 days a week for 3 weeks; active exercises: 9 sessions, 3 days a week for 3 weeks; home based exercises: NR; education: 60 min, 1 sessions.	Interferential currents + active exercises + home based exercises + education. Interferential currents: 20 min, 15 sessions, 5 days a week for 3 weeks; active exercises: 9 sessions, 3 days a week for 3 weeks; home based exercises: NR; education: 60 min, 1 sessions.	Sham SWD + active exercises + home based exercises + education. Sham SWD: 15 sessions, 5 days a week for 3 weeks; active exercises: 9 sessions, 3 days a week for 3 weeks; home based exercises: NR; education: 60 min, 1 sessions.
Bezalel 2010	Active exercises + education. 45 min, 4 sessions, once a week for 4 weeks.				

AUTHOR & YEAR	CONTROL I	CONTROL II	CONTROL III	CONTROL IV	CONTROL V
Boyaci 2013	Ultrasound. 8 min each knee, 10 sessions, 5 days a week for 2 weeks.	Phonophoresis (ketoprofen) [ultrasound + topical application of ketoprofen to enhance absoption]. 8 min each knee, 10 sessions, 5 days a week for 2 weeks.			
Callaghan 2005	Sham SWD. 20 min, 6 sessions, 3 days a weeks for 2 weeks.				
Cantarini 2006	Mud pack + hot water baths. Mud pack: 20 min, 15 sessions, for 3 weeks; hot water baths: 15 min, 15 sessions, for 3 weeks.	Regular routine ambulatory care. NR.			
Cetin 2008	TENS + hot pack + active exercises . TENS: 20 min, 24 sessions, 3 days a week for 8 weeks; hot packs and exercise: 24 sessions, 3 days a week for 8 weeks.	Ultrasound + hot pack + active exercises. Ultrasound: 10 min, 24 sessions, 3 days a week for 8 weeks; hot packs and exercise: 24 sessions, 3 days a week for 8 weeks.	Hot pack + active exercises. 24 sessions, 3 days a week for 8 weeks.		
Chamberlain 1982	Active exercises. 6 sessions, 3 days a week for 2 weeks, then exercise at home.				
Clarke 1974	Ice. 9 sessions, 3 days a week for 3 weeks.	Sham SWD. 9 sessions, 3 days a week for 3 weeks.			
Fukuda 2011	No treatment	Sham SWD. 19 min, 9 sessions, 3 days a week for 3 weeks.			
Fukuda 2008	No treatment	Sham SWD. 19 min, 9 sessions, 3 days a week for 3 weeks.			

AUTHOR & YEAR	CONTROL I	CONTROL II	CONTROL III	CONTROL IV	CONTROL V
Gomes 2020	Active exercises. 90 min, 24 sessions, 3 days a week for 8 weeks.	Interferential currents + active exercises. Interferential currents: 40 min, 24 sessions, 3 days a week for 8 weeks; active exercises: 90 min, 24 sessions, 3 days a week for 8 weeks.	Photobiomodulation + active exercises. Photobiomodulation: 24 sessions, 3 days a week for 8 weeks; active exercises: 90 min, 24 sessions, 3 days a week for 8 weeks.	Sham ultrasound + active exercises. Sham ultrasound: 20 min, 24 sessions, 3 days a week for 8 weeks; active exercises: 90 min, 24 sessions, 3 days a week for 8 weeks.	
Işik 2020	Sham SWD + Injection + home based active exrcises. Sham SWD and injection: 20 min, 3 sessions, once a week for 3 weeks; home-based exercise: 40 min, 36 sessions, 6 days a week for 6 weeks.				
Jia 2022	Ultrasound. 20 min, 12 sessions, once a day.				
Klaber Moffett 1996/1994	No treatment	Sham SWD. 15 min, 9 sessions, 3 days a week for 3 weeks.			
Ovanessian 2008	No treatment				
Rattanachaiyanont 2008	Sham SWD + active exercises + education. Sham SWD: 20 min, 9 sessions, 3 days a week for 3 weeks. Active exercise and education: NR.				

AUTHOR & YEAR	CONTROL I	CONTROL II	CONTROL III	CONTROL IV	CONTROL V
Terzi 2017	Ultrasound + education + active exercises + TENS + hot pack. Ultrasound: 5 min, 10 sessions, 5 days a week for 2 weeks; education: NR; active exercises: 84 at home sessions, 14 times a week (twice a day) for 6 weeks; TENS: 20 min, 10 sessions, 5 days a week for 2 weeks; hot packs: 20 min, 10 sessions, 5 days a week for 2 weeks.	Education + active exercises. Education: NR; active exercises: 84 at home sessions, 14 times a week (twice a day) for 6 weeks.			
Teslim 2013					
Tüzün 2003					
Wright 1964	Placebo tablets (twice a day). NR.	4 Placebo injections. NR.			

AUTHOR & YEAR	CONTROL I	CONTROL II	CONTROL III	CONTROL IV	CONTROL V
Giombini 2010	Sham MWD. 30 min, 12 sessions, 3 days a week for 4 weeks.				
Rabini 2012	Superficial heat therapy. 30 min, 12 sessions, 3 days a week for 4 weeks.				
Alcidi 2007	TENS. 20 min, 5 sessions, 5 days for 1 weeks.				
Cocetta 2018	Sham CRET. 20 min (5 min of detuned capacitive, 10 min of detuned resistive, 5 min of detuned capacitive), 6 sessions, 3 days a week for 2 weeks.				
Kumaran 2019	Home based exercise + education. 3 sessions in 4 weeks.	Sham CRET + home based exercises + education. Sham CRET: 15 minutes, 8 sessions, 2 days a week for 4 weeks; home based exercise + education: 3 sessions in 4 weeks.			
Ahmed 2009	Sham SWD + drug + active exercises + education. Sham SWD: 15 min, 18 sessions, 3 days a week for 6 weeks; drug: 7 days a week; active exercise: NR; education: NR.				
Ansari 2022	Massage. 20 min, 10 sessions, on alternate days for 3 weeks.				
Gibson 1985	Mobilisation. 4 sessions, once a week for 4 weeks.	Sham SWD. 12 sessions, 3 days a week for 4 weeks.			

AUTHOR & YEAR	CONTROL I	CONTROL II	CONTROL III	CONTROL IV	CONTROL V
Igatpurkar 2013	Mobilisation + hot pack + active exercises. 4 weeks of treatment.				
Kumar 2009	Active exercises. 4 sessions, once a week for 4 weeks.				
Kumar 2009a	Active exercises. 4 sessions, once a week for 4 weeks.				
Shakoor 2008	Sham SWD + drug + active exercises + education. Sham SWD: 15 min, 18 sessions, 3 days a week for 6 weeks; drug: twice a day 7 days a week; active exercise: 84 sessions, twice a day 7 days a week for 6 weeks; education: NR.				
Durmus 2014	Active exercises. 60 min, 3 days a week for 6 weeks.				

AUTHOR & YEAR	CONTROL I	CONTROL II	CONTROL III	CONTROL IV	CONTROL V
Farrell 1982	Mobilisation. 9 sessions, 3 days a week for 3 weeks.				
Notarnicola 2017	Laser therapy. 10 sessions, 5 days a week for 2 weeks.				
Wachi 2022	Sham CRET. NR.				
Zati 2018					
Jiménez-Garcia 2008	Ultrasound + iontophoresis with acetic acid + active exercises. Ultrasound: 5 min, 15 sessions, 5 days a week for 3 weeks; iontophoresis with acetic acid: 15 min, 15 sessions, 5 days a week for 3 weeks; active exercise: 15 sessions, 5 days a week for 3 weeks.				
Yilmaz 2018	Sham SWD + active exercises + cold pack + drug. Sham SWD: 20 min, 10 sessions, 5 days a week for 2 weeks; active exerise: NR; cold pack: 20 min, 3 times a day; drug: 2 weeks.	Sham SWD + active exercises + cold pack + drug. Sham SWD: 20 min, 10 sessions, 5 days a week for 2 weeks; active exerise: NR; cold pack: 20 min, 3 times a day; drug: 2 weeks.			

AUTHOR & YEAR	CONTROL I	CONTROL II	CONTROL III	CONTROL IV	CONTROL V
Akyol 2012	Sham MWD + hot pack + active exercises. Sham MWD: 20 min, 15 sessions, 5 days a week for 3 weeks; hot packs: 20 min, 15 sessions, 5 days a week for 3 weeks; active exercises: 15 min, 15 sessions, 5 days a week for 3 weeks.				
Giombini 2006	Ultrasound. 15 min, 12 sessions, 3 days s a week for 4 weeks.	Active exercises. 5 min twice a day, 56 sessions, 7 days a week for 4 weeks.			
Rabini 2012	Local injections. 3 sessions, 1 injection every 2 weeks.				
Avendaño-Coy 2022	Sham SCRET + exercises. Sham CRET: 15 min, 9 sessions, 3 days a week for 3 weeks; exercises: 15 sessions, 5 days a week for 3 weeks.				
Guler-Uysal 2004	Mobilization + active exercises. Mobilization: NR; active exercises: NR.				
Leung 2008	Hot pack + stretching. Hot pack: 20 min; stretching: 12 sessions, 3 days a week for 4 weeks.	Stretching: 12 sessions, 3 days a week for 4 weeks.			
Hammad 2019	Mobilization: 9 sessions, 3 days a week for 3 weeks.				

AUTHOR & YEAR	CONTROL I	CONTROL II	CONTROL III	CONTROL IV	CONTROL V
Boyaci 2014	Sham SWD. 20 min, 15 sessions, 5 days a week for 3 weeks.				
Incebiyik 2015	Sham SWD + hot pack + nerve gliding exercises. Sham SWD: 15 min, 15 sessions, 5 days a week for 3 weeks; hot pack: 15 min, 15 sessions, 5 days a week for 3 weeks; nerve gliding exercises: 15 sessions, 5 days a week for 3 weeks.				
Frasca 2011	Sham MWD. 20 min, 6 sessions, 2 days a week for 3 weeks.				
Cheng 2018	ESWT (extracorporeal shock wave therapy): 16 sessions, once a week for 16 weeks.				
Giombini 2002	Ultrasound. 15 min, 12 sessions, 3 days s a week for 4 weeks.				
Dziedzic 2005	Active exercise + education. 20 min, 8 sessions, 6 weeks.	Manual therapy + education. 20 min, 8 sessions, 6 weeks.			
Ortega 2013	Sham MWD + active exercises + TENS. 15 sessions, 5 days a week for 3 weeks.				
Verma 2012	Taping + active exercises. Taping: 2 weeks on alternate days; active exercise: NR.				

AUTHOR & YEAR	CONTROL I	CONTROL II	CONTROL III	CONTROL IV	CONTROL V
Albornoz-Cabello 2020	Active exercises. 10 min, 10 sessions, 5/3/2 days a weeks progressively for 3 weeks.				
Gray 1995	Megapulse. 20 min, 12 sessions, 3 days a week for 4 weeks.	Ultrasound. 2 min, 12 sessions, 3 days a week for 4 weeks.	Laser. 3 min, 12 sessions, 3 days a week for 4 weeks.	Sham treatment. 12 sessions, 3 days a week for 4 weeks.	
Talaat 1986	Drug. 7 tablets, once a day for 1 week.	Ultrasound. 5 min, 14 sessions, 7 days a week for 2 weeks.			
Nakamura 2022	No intervention.				
Visconti 2020	Massage. 10 min.	Sham CRET. 10 min.			
Livesley 1992	Sham SWD + standard physiotherapy. NR.				
Babaei-Ghazani 2019	Sham SWD + massage + stretching + active exercises + education. Sham SWD: 15 min, 10 sessions; massage + stretching + active exercises + education: NR.				
Badur 2020	Sham SWD. 20 min, 10 sessions, 5 days a week for 2 weeks.				
Pasila 1978	Sham SWD. NR.				
Giombini 2001	Ultrasound. 15 min, 9 sessions, 3 days a week for 3 weeks.				

AUTHOR & YEAR	CONTROL I	CONTROL II	CONTROL III	CONTROL IV	CONTROL V
Georgoudis 2017	Acupuncture + stretching. Acupuncture: 20 min, 10 sessions; stretching: NR, 2-3 times daily.				
García-Marín 2021	Active exercises. 30 min, 10 sessions, 5 days a week for 2 weeks.	Sham LWD + active exercises. Sham LWD: 12 min, 10 sessions, 5 days a week for 2 weeks; active exercises: 30 min, 10 sessions, 5 days a week for 2 weeks.			

AUTHOR & YEAR	PARTICIPANTS INTERVENTION GROUP I (M:F)	PARTICIPANTS INTERVENTION GROUP II (M:F)	PARTICIPANTS INTERVENTION GROUP III (M:F)	PARTICIPANTS INTERVENTION GROUP IV (M:F)	PARTICIPANTS INTERVENTION GROUP V (M:F)	PARTICIPANTS CONTROL GROUP I (M:F)	PARTICIPANTS CONTROL GROUP II (M:F)	PARTICIPANTS CONTROL GROUP III (M:F)	PARTICIPANTS CONTROL GROUP IV (M:F)	PARTICIPANTS CONTROL GROUP V (M:F)
Akyol 2010	20:0					20:0				
Atamaz 2006	6:34					2:18	5:15			
Atamaz 2012	4:27					10:27	6:31	7:28	4:27	5:27
Bezalel 2010	5:20					8:17				

AUTHOR & YEAR	PARTICIPANTS INTERVENTION GROUP I (M:F)	PARTICIPANTS INTERVENTION GROUP II (M:F)	PARTICIPANTS INTERVENTION GROUP III (M:F)	PARTICIPANTS INTERVENTION GROUP IV (M:F)	PARTICIPANTS INTERVENTION GROUP V (M:F)	PARTICIPANTS CONTROL GROUP I (M:F)	PARTICIPANTS CONTROL GROUP II (M:F)	PARTICIPANTS CONTROL GROUP III (M:F)	PARTICIPANTS CONTROL GROUP IV (M:F)	PARTICIPANTS CONTROL GROUP V (M:F)
Boyaci 2013	0:35					0:33	0:33			
Callaghan 2005	5:4	4:5				5:4				
Cantarini 2006	8:16					13:17	6:14			
Cetin 2008	0:20					0:20	0:20	0:20		
Chamberlain 1982	6:16					4:16				
Clarke 1974	6:11					4:11	4:9			
Fukuda 2011	0:31	0:32				0:35	0:23			
Fukuda 2008	24 (M:F NR)	22 (M:F NR)				18 (M:F NR)	20 (M:F NR)			

AUTHOR & YEAR	PARTICIPANTS INTERVENTION GROUP I (M:F)	PARTICIPANTS INTERVENTION GROUP II (M:F)	PARTICIPANTS INTERVENTION GROUP III (M:F)	PARTICIPANTS INTERVENTION GROUP IV (M:F)	PARTICIPANTS INTERVENTION GROUP V (M:F)	PARTICIPANTS CONTROL GROUP I (M:F)	PARTICIPANTS CONTROL GROUP II (M:F)	PARTICIPANTS CONTROL GROUP III (M:F)	PARTICIPANTS CONTROL GROUP IV (M:F)	PARTICIPANTS CONTROL GROUP V (M:F)
Gomes 2020	1:19					3:17	2:18	0:20	2:18	
Işik 2020	14:17					13:19				
Jia 2022	12:45					15:42				
Klaber Moffett 1996/1994	34:58					34:58	34:58			
Ovanessian 2008	14 (M:F NR)	14 (M:F NR)				14 (M:F NR)				
Rattanachaiyanont 2008	0:53					0:60				

AUTHOR & YEAR	PARTICIPANTS INTERVENTION GROUP I (M:F)	PARTICIPANTS INTERVENTION GROUP II (M:F)	PARTICIPANTS INTERVENTION GROUP III (M:F)	PARTICIPANTS INTERVENTION GROUP IV (M:F)	PARTICIPANTS INTERVENTION GROUP V (M:F)	PARTICIPANTS CONTROL GROUP I (M:F)	PARTICIPANTS CONTROL GROUP II (M:F)	PARTICIPANTS CONTROL GROUP III (M:F)	PARTICIPANTS CONTROL GROUP IV (M:F)	PARTICIPANTS CONTROL GROUP V (M:F)
Terzi 2017	13:25					13:26	11:19			
Teslim 2013	12 (M:F NR)	12 (M:F NR)								
Tüzün 2003	7:13	7:13								
Wright 1964	1:12					0:13	2:10			

AUTHOR & YEAR	PARTICIPANTS INTERVENTION GROUP I (M:F)	PARTICIPANTS INTERVENTION GROUP II (M:F)	PARTICIPANTS INTERVENTION GROUP III (M:F)	PARTICIPANTS INTERVENTION GROUP IV (M:F)	PARTICIPANTS INTERVENTION GROUP V (M:F)	PARTICIPANTS CONTROL GROUP I (M:F)	PARTICIPANTS CONTROL GROUP II (M:F)	PARTICIPANTS CONTROL GROUP III (M:F)	PARTICIPANTS CONTROL GROUP IV (M:F)	PARTICIPANTS CONTROL GROUP V (M:F)
Giombini 2010	12:23					9:19				
Rabini 2012	4:23					5:22				
Alcidi 2007	2:18					4:16				
Cocetta 2018	6:25					0:22				
Kumaran 2019	6:9					6:9	6:9			
Ahmed 2009	64:46					64:46				
Ansari 2022	24 (M:F NR)					24 (M:F NR)				
Gibson 1985	18:16					20:21	23:11			

AUTHOR & YEAR	PARTICIPANTS INTERVENTION GROUP I (M:F)	PARTICIPANTS INTERVENTION GROUP II (M:F)	PARTICIPANTS INTERVENTION GROUP III (M:F)	PARTICIPANTS INTERVENTION GROUP IV (M:F)	PARTICIPANTS INTERVENTION GROUP V (M:F)	PARTICIPANTS CONTROL GROUP I (M:F)	PARTICIPANTS CONTROL GROUP II (M:F)	PARTICIPANTS CONTROL GROUP III (M:F)	PARTICIPANTS CONTROL GROUP IV (M:F)	PARTICIPANTS CONTROL GROUP V (M:F)
Igatpurkar 2013	7:13					6:14				
Kumar 2009	15:0					15:0				
Kumar 2009a	51:0					51:0				
Shakoor 2008	42:60					42:60				
Durmus 2014	0:19					0:20				

AUTHOR & YEAR	PARTICIPANTS INTERVENTION GROUP I (M:F)	PARTICIPANTS INTERVENTION GROUP II (M:F)	PARTICIPANTS INTERVENTION GROUP III (M:F)	PARTICIPANTS INTERVENTION GROUP IV (M:F)	PARTICIPANTS INTERVENTION GROUP V (M:F)	PARTICIPANTS CONTROL GROUP I (M:F)	PARTICIPANTS CONTROL GROUP II (M:F)	PARTICIPANTS CONTROL GROUP III (M:F)	PARTICIPANTS CONTROL GROUP IV (M:F)	PARTICIPANTS CONTROL GROUP V (M:F)
Farrell 1982	14:10					16:8				
Notarnicola 2017	17:43					17:43				
Wachi 2022	15:0					15:0				
Zati 2018	10:14	13:12								
Jiménez-Garcia 2008	2:10					1:10				
Yilmaz 2018	5:9	4:10				0:15	4:10			

AUTHOR & YEAR	PARTICIPANTS INTERVENTION GROUP I (M:F)	PARTICIPANTS INTERVENTION GROUP II (M:F)	PARTICIPANTS INTERVENTION GROUP III (M:F)	PARTICIPANTS INTERVENTION GROUP IV (M:F)	PARTICIPANTS INTERVENTION GROUP V (M:F)	PARTICIPANTS CONTROL GROUP I (M:F)	PARTICIPANTS CONTROL GROUP II (M:F)	PARTICIPANTS CONTROL GROUP III (M:F)	PARTICIPANTS CONTROL GROUP IV (M:F)	PARTICIPANTS CONTROL GROUP V (M:F)
Akyol 2012	15:5					15:5				
Giombini 2006	12:2					8:4	9:2			
Rabini 2012	16:30					15:31				
Avendaño-Coy 2022	10:17	10:17				9:18				
Guler-Uysal 2004	7:13					5:15				
Leung 2008	5:5					8:2	8:2			
Hammad 2019	7:8					8:7				

AUTHOR & YEAR	PARTICIPANTS INTERVENTION GROUP I (M:F)	PARTICIPANTS INTERVENTION GROUP II (M:F)	PARTICIPANTS INTERVENTION GROUP III (M:F)	PARTICIPANTS INTERVENTION GROUP IV (M:F)	PARTICIPANTS INTERVENTION GROUP V (M:F)	PARTICIPANTS CONTROL GROUP I (M:F)	PARTICIPANTS CONTROL GROUP II (M:F)	PARTICIPANTS CONTROL GROUP III (M:F)	PARTICIPANTS CONTROL GROUP IV (M:F)	PARTICIPANTS CONTROL GROUP V (M:F)
Boyaci 2014	2:8	1:9				1:9				
Incebiyik 2015	0:15					0:13				
Frasca 2011	2:9					1:10				
Cheng 2018	13:12					12:14				
Giombini 2002	33:11					33:11				
Dziedzic 2005	40:81					52:63	37:77			
Ortega 2013	13:37	10:38				13:38				
Verma 2012	10 (M:F NR)					10 (M:F NR)				

AUTHOR & YEAR	PARTICIPANTS INTERVENTION GROUP I (M:F)	PARTICIPANTS INTERVENTION GROUP II (M:F)	PARTICIPANTS INTERVENTION GROUP III (M:F)	PARTICIPANTS INTERVENTION GROUP IV (M:F)	PARTICIPANTS INTERVENTION GROUP V (M:F)	PARTICIPANTS CONTROL GROUP I (M:F)	PARTICIPANTS CONTROL GROUP II (M:F)	PARTICIPANTS CONTROL GROUP III (M:F)	PARTICIPANTS CONTROL GROUP IV (M:F)	PARTICIPANTS CONTROL GROUP V (M:F)
Albornoz-Cabello 2020	32:52					32:52				
Gray 1995	1:6					1:6	1:6	1:6	1:6	
Talaat 1986	76:44					76:44	76:44			
Nakamura 2022	15:0					13:0				
Visconti 2020	55:0					55:0	55:0			
Livesley 1992	5:17					6:20				
Babaei-Ghazani 2019	8:17					12:13				
Badur 2020	29:32					29:32				
Pasila 1978	50:50	51:49				51:49				
Giombini 2001	29:11					29:11				

AUTHOR & YEAR	PARTICIPANTS INTERVENTION GROUP I (M:F)	PARTICIPANTS INTERVENTION GROUP II (M:F)	PARTICIPANTS INTERVENTION GROUP III (M:F)	PARTICIPANTS INTERVENTION GROUP IV (M:F)	PARTICIPANTS INTERVENTION GROUP V (M:F)	PARTICIPANTS CONTROL GROUP I (M:F)	PARTICIPANTS CONTROL GROUP II (M:F)	PARTICIPANTS CONTROL GROUP III (M:F)	PARTICIPANTS CONTROL GROUP IV (M:F)	PARTICIPANTS CONTROL GROUP V (M:F)
Georgoudis 2017	24 (M:F NR)					20 (M:F NR)				
García-Marín 2021	6:8					5:9	4:10			
